# GIRK1 triggers multiple cancer-related pathways in the benign mammary epithelial cell line MCF10A

**DOI:** 10.1038/s41598-019-55683-w

**Published:** 2019-12-17

**Authors:** Gebhard Schratter, Susanne Scheruebel, Sonja Langthaler, Katja Ester, Brigitte Pelzmann, Nassim Ghaffari-Tabrizi-Wizsy, Simin Rezania, Astrid Gorischek, Dieter Platzer, Klaus Zorn-Pauly, Helmut Ahammer, Andreas Prokesch, Stefanie Stanzer, Trevor T. J. Devaney, Kurt Schmidt, Stephan W. Jahn, Ruth Prassl, Thomas Bauernhofer, Wolfgang Schreibmayer

**Affiliations:** 1 Gottfried Schatz Research Center for Cell Signaling, Metabolism and Aging, Medical University of Graz, Graz, Austria; 2 Institute for Health Care Engineering with European Testing Center of Medical Devices, Graz University of Technology, Graz, Austria; 3 Laboratory of Experimental Therapy, Division of Molecular Medicine, Ruđer Bošković Institute, Bijenička 54, 10000 Zagreb, Croatia; 4 Institute of Immunology and Pathophysiology, SFL Chicken CAM Laboratory, Medical University of Graz, Graz, Austria; 5 Cell Biology, Histology and Embryology, Medical University of Graz, Graz, Austria; 6 Division of Oncology, Department of Internal Medicine, Medical University of Graz, Graz, Austria; 7 Institute of Pharmaceutical Sciences, Karl Franzens University of Graz, Graz, Austria; 8 Diagnostic & Research Institute of Pathology, Medical University of Graz, Graz, Austria; 9 Research Unit on Ion Channels and Cancer Biology, Medical University of Graz, Graz, Austria; 10 BioTechMed-Graz, 8010 Graz, Austria

**Keywords:** Biochemistry, Biophysics, Cancer, Cell biology, Molecular biology, Biomarkers, Diseases, Medical research, Molecular medicine, Oncology

## Abstract

Excessive expression of subunit 1 of GIRK1 in ER^+^ breast tumors is associated with reduced survival times and increased lymph node metastasis in patients. To investigate possible tumor-initiating properties, benign MCF10A and malign MCF7 mammary epithelial cells were engineered to overexpress GIRK1 neoplasia associated vital parameters and resting potentials were measured and compared to controls. The presence of GIRK1 resulted in resting potentials negative to the controls. Upon GIRK1 overexpression, several cellular pathways were regulated towards pro-tumorigenic action as revealed by comparison of transcriptomes of MCF10A^GIRK1^ with the control (MCF10A^eGFP^). According to transcriptome analysis, cellular migration was promoted while wound healing and extracellular matrix interactions were impaired. Vital parameters in MCF7 cells were affected akin the benign MCF10A lines, but to a lesser extent. Thus, GIRK1 regulated cellular pathways in mammary epithelial cells are likely to contribute to the development and progression of breast cancer.

## Introduction

G-Protein coupled receptors (GPCRs) represent one of the largest gene families in humans and comprise a sizeable number of plasma membrane bound sensors as cellular targets for endocrine signaling molecules. Opposed to this stands a handful of direct effectors for G-protein signaling. Amongst these effectors are G-Protein-activated Inwardly Rectifying Potassium Channels (GIRKs) which link the presence of extracellular signalling molecules to hyperpolarisation of the membrane potential. Activation of GIRKs occurs following the binding of ligands to GPCRs. Subsequently, membrane delimited pertussis toxin-sensitive G-protein βγ subunits are released and directly activate GIRK K^+^ channels, stabilizing the cells’ resting membrane potential. Four different genes exist in the human genome which encode GIRK1 (Kir3.1), GIRK2 (Kir3.2), GIRK3 (Kir3.3) and GIRK4 (Kir3.4). These subunits form heterotetrameric and/or homotetrameric ion channels in the plasma membrane^1^. GIRKs serve important physiological functions in the nervous system, heart, pancreas, blood platelets and in the regulation of lipid metabolism in fat cells^2–8^. Connections between inherited and/or somatic mutations in genes encoding GIRKs and neuronal, endocrine and cardiac disorders have been established, underlining the impact of this inhibitory G-protein pathway for physiological as well as pathological processes in humans^1,9,10^. Most notably, an abnormal and pronounced overexpression of GIRK1 occurs in a subset of estrogen receptor positive (ER^+^) primary breast tumors, whereas in healthy breast tissue GIRK1 was practically undetectable^11^. Excessive protein and mRNA levels of GIRK1 come along with an increase of lymph node metastasis and reduced survival of patients^12–14^. Therefore, GIRK1 poses a promising target for prognosis and therapy in breast cancer. Substantial expression of GIRK1 mRNA and protein also occurs in several cell lines cultured from breast tumors^15,16^. The malignant MCF7 breast cancer cell line expresses GIRK1 mRNA already at moderate levels, thus providing an excellent model for increased GIRK1 expression in breast tumours^17^. Ectopic hyperexpression of GIRK1 in MCF7 has been shown to aggravate several cancer hallmarks, including increased cellular motility, invasiveness and angiogenesis, thereby providing an explanation for the observed impact under clinical conditions^18^. In order to elucidate whether GIRK1 can also exhibit tumor-initiating properties in healthy tissue, we overexpressed GIRK1 in the benign MCF10A mammary epithelial cell (MEC) line that possesses marginal endogenous GIRK1 levels. In order to identify cellular pathways altered by GIRK1 overexpression, we interrogated transcriptome wide expression changes, induced by stable GIRK1 overexpression, in MCF10A cells. Vital assays and electrophysiological properties of the different MCF10A based lines generated were compared to each other. The results indicated that divergent biological properties correlated with differentially expressed pathways derived from transcriptome analysis. In addition, MEC lines based upon the malignant MCF7 line were engineered. Their vital properties were assessed and the effects of GIRK1 overexpression was compared to the benign MEC line.

## Results

### Validation and characterization of MEC lines overexpressing GIRK1

**Figure 1:**
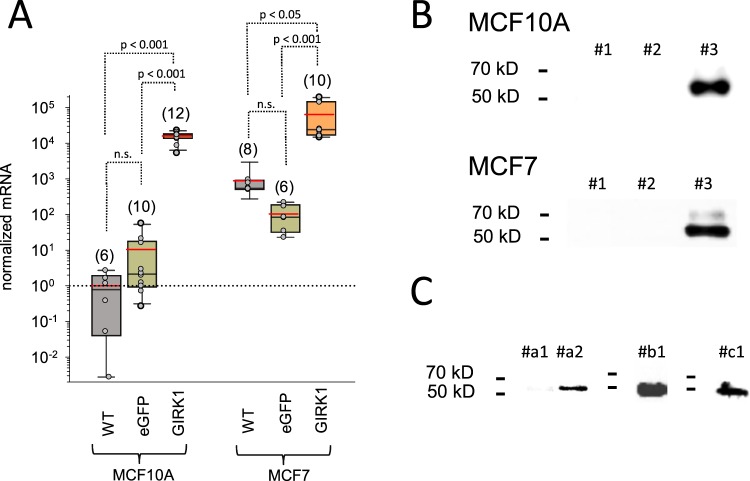
Quantification of GIRK1 mRNA and protein in the different cell lines. (**A**) GIRK1 mRNA levels normalized to MCF10A^**WT**^. WT, eGFP and GIRK1 denote wildtype, eGFP (control) and GIRK1 overexpressors, respectively. The median value is represented by the black line within the box, box margins represent 75% and 25% percentiles, whiskers indicate 90% and 10% percentiles. The red line represents the mean value. Individual values are shown as grey circles. The number of individual experiments is given in parenthesis above each box. Statistically significant differences between groups are indicated above brackets (n.s.: statistically not significant). (**B**) WB analysis of cell lysates: *upper panel*: MCF10A lysates (3 µg protein per slot was applied) probed with GIRK1^CT^ Ab. #1: MCF10A^WT^, #2: MCF10A^eGFP^ and #3: MCF10A^GIRK1^. *Lower panel*: MCF7 lysates (3 µg protein per slot) probed with GIRK1^CT^ Ab. #1: MCF7^WT^, #2: MCF7^eGFP^ and #3: MCF7^GIRK1^. (**C**)WB analysis of immunoprecipitates derived using GIRK1^CT^ Ab on MCF7^WT^ cell lysates. *Left*: membrane probed using GIRK1^CT^ Ab. #a1: lysate (30 µl), #a2: IP (30 µl). Middle: #b1: IP (30 µl) membrane probed using GIRK1^NT^ Ab (N-terminal) Ab. Right: #c1: IP (30 µl). Membrane probed using monoclonal GIRK1 Ab.

First, mRNA levels in the MEC lines generated were assessed by RT-qPCR (Fig. 1A). Expression of mRNA encoding GIRK1 was increased more than four orders of magnitude in MCF10A^GIRK1^ cells when compared to MCF10A^WT^, where GIRK1 mRNA was barely detectable (observed C_T_ value as measured by qPCR was 35.26 ± 1.32 (mean ± SEM); N = 6). In comparison GIRK1 mRNA levels were more than two orders of magnitude higher in the malign MCF7^WT^ vs. the benign MCF10A^WT^ (p < 0.001). Overexpression of GIRK1 caused a further increase of mRNA levels by two orders of magnitude in MCF7^GIRK1^ when compared to MCF7^WT^. mRNA expression levels of control cell lines (MCF10A^eGFP^ & MCF7^eGFP^) resembled the respective wild type lines. Secondly, GIRK1 protein expression was assessed by Western blot (WB) analysis (Fig. 1B). GIRK1 protein was clearly detected in both MCF10A^GIRK1^ and MCF7^GIRK1^ lines by Western Blot (WB) analysis, while being barely detectable in the corresponding WT and control lines. Although mRNA levels encoding GIRK1 were substantial in MCF7^WT^, corresponding protein amounts could not be reliably detected by WB. Immunoprecipitation (IP) was performed on MCF7^WT^ using antibody (Ab) directed against GIRK1 C-terminus (C-T; Fig. 1C). GIRK1 protein was not only detected in the precipitate when tested with GIRK1^CT^ Ab, but also with GIRK1^NT^ Ab or monoclonal GIRK1 Ab that recognize different epitopes on the GIRK1 protein. In summary, we could verify that the MCF10A^GIRK1^ and MCF7^GIRK1^ transfectant cell lines exhibit relevant overexpression of GIRK1 mRNA and protein compared to the respective WT lines. In addition, GIRK1 mRNA and protein levels are substantial in the malign estrogen receptor positive MCF7^WT^ line compared to the benign MCF10A^WT^ MEC line. Thus, the set of cell lines generated pose a plausible model to study functional effects of elevated GIRK1 levels in benign and malignant MECs.

### GIRK1 overexpression produces functional GIRK channels both in MCF10A and MCF7 based MEC lines

**Figure 2:**
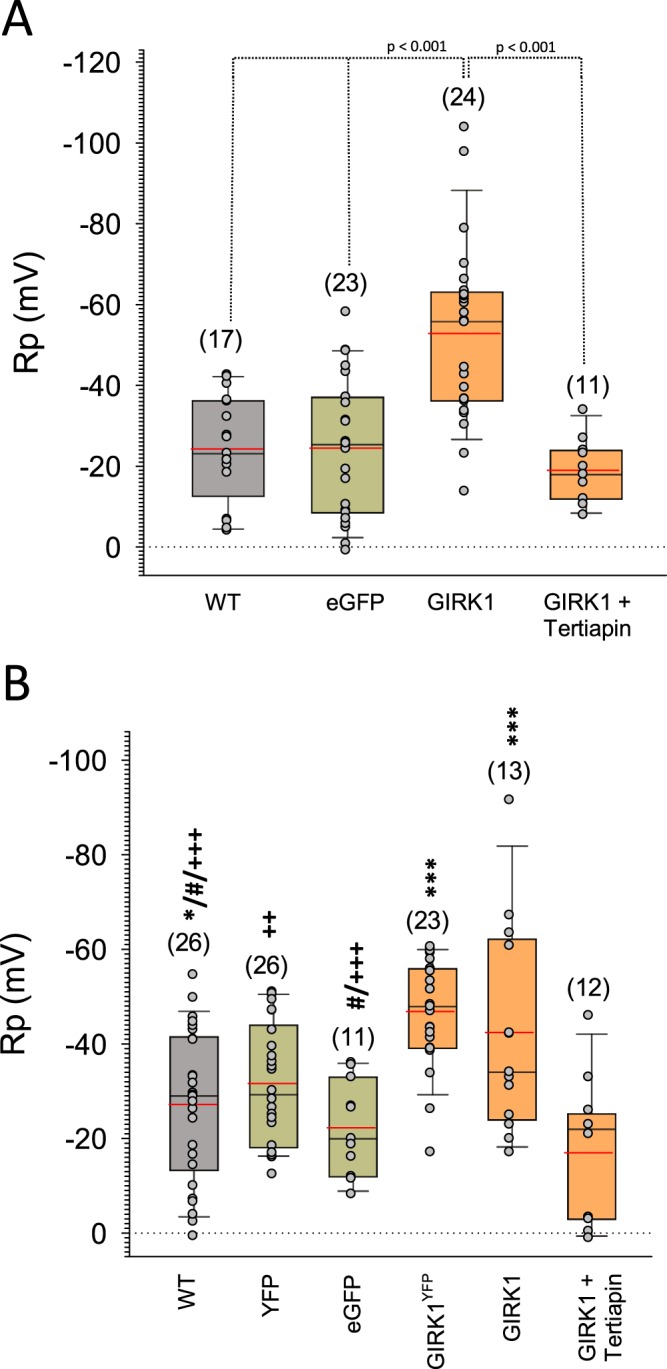
Membrane resting potentials in the different MEC lines. (**A**) Membrane resting potentials of MCF10A cells. *WT:* MCF10A^WT^, *eGFP:* MCF10A^eGFP^, *GIRK1:* MCF10A^GIRK1^ and *GIRK1* + *Tertiapin:* MCF10A^GIRK1^ treated with 200 nmole/L tertiapin-Q. (**B**) Membrane resting potentials of MCF7 cells. *WT:* MCF7^WT^, *eYFP:* MCF7^eYFP^, *eGFP:* MCF7A^eGFP^, *GIRK1*^*eYFP*^*:* MCF7^GIRK1/eYFP^, *GIRK1:* MCF7^GIRK1^ and *GIRK1* + *Tertiapin:* MCF7^GIRK1^ treated with 200 nmole/L tertiapin-Q. Number of experiments is given in parenthesis above each bar. *,(***): The group differs statistically significant from *GIRK1* + *Tertiapin* at the p < 0.05 (<0.001) level. #: The group differs statistically significant from *GIRK1* at the p < 0.05 level. + , (++,+++): The group differs statistically significant from *GIRK1*^*eYFP*^ at the p < 0.05 (<0.01, <0.001) level.

In MCF10A^GIRK1^ membrane resting potentials (RPs) were −52.8 ± 4.5 mV (mean value ± SEM) and hyperpolarized when compared to controls that ranged around −24 mV (MCF10A^WT^: −24.3 ± 3.2 mV, MCF10A^eGFP^: −24.5 ± 3.6 mV; Fig. 2A). In the presence of 200 nmol/L tertiapin-Q, a specific blocker of GIRK1 containing GIRK channels^19,20^, RPs approached and exceeded control values (−19.0 ± 2.3 mV), indicating that overexpressed GIRK1 protein produces functional K^+^ channels in MCF10A. RPs were assessed in the MCF7 based cell lines described here and, in addition, in MCF7 based cell lines, stably overexpressing chimeric GIRK1/eYFP protein, described previously (^18^; Fig. 2B (see Supplementary Figure 1 for GIRK1 mRNA levels of chimeric vs. bicistronic MCF7 lines)). Both MCF7^GIRK1^ and MCF7^GIRK1/eYFP^ exerted hyperpolarized RPs when compared to respective controls, albeit the differences in RPs between overexpressors and control groups were less pronounced in comparison to benign MCF10A lines. Moreover, when GIRK channels were blocked by tertiapin-Q in MCF7^GIRK1^, RPs with −17.0 ± 4.3 mV were the most depolarized among MCF7 based lines and considerably higher when compared to MCF7^WT^ (−27.2 ± 3.1 mV). These observations demonstrate that functional GIRK channels formed upon overexpression of GIRK1 in the malignant MCF7 and in the benign MCF10A MEC lines. In addition, minute amounts of functional GIRK channel complexes already preexist in MCF7^WT^, corresponding to the moderate GIRK1 mRNA and protein levels.

### GIRK1 overexpression triggers several pro-tumorigenic pathways in benign MECs

**Figure 3:**
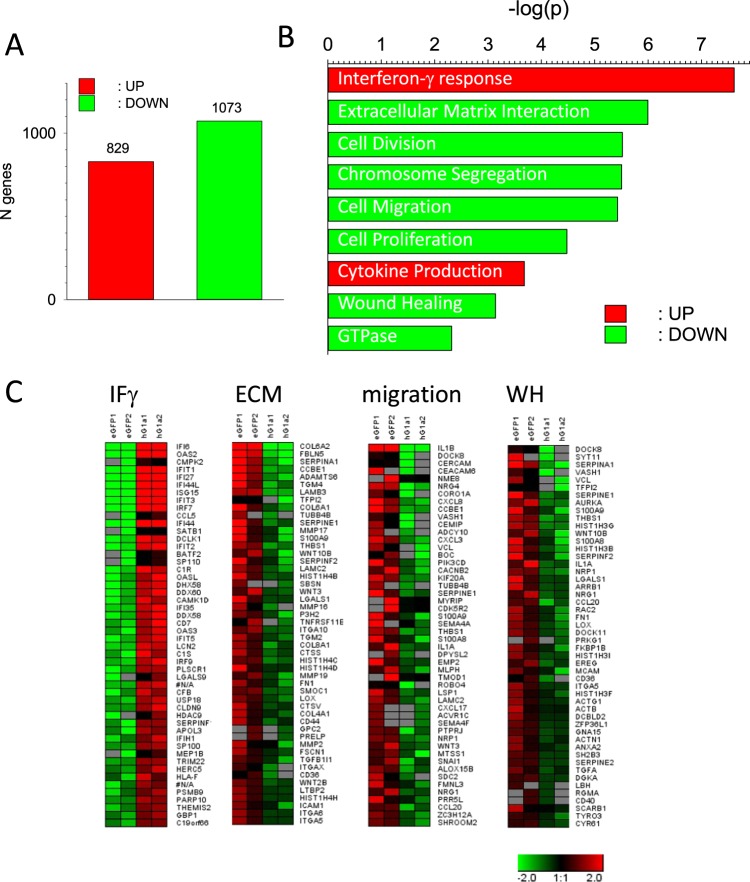
Effect of GIRK1 overexpression on transcriptome of MCF10A cells. Number of significantly up- or downregulated transcripts when MCF10A^eGFP^ are compared to MCF10A^GIRK1^. *Red:* upregulated transcripts, *green:* downregulated transcripts. (**A**) Top nine gene ontology clusters derived by DAVID functional clustering. (**B**) Heat maps displaying the fold changes of expression levels of the top 50 genes of selected GO terms. *IF* γ*:* Interferon-γ response. *ECM:* extracellular matrix interaction. *Migration:* cell migration and *WH:* wound healing. *Bottom right:* color coding for the log2 fold change.

In order to identify a possible cancerogenic influence of GIRK1 overexpression in benign MECs, transcriptomes of MCF10A^GIRK1^ were compared to the ones of MCF10A^eGFP^. Unexpected for the overexpression of a single K^+^ channel subunit, a high number of transcripts were sizably up- or downregulated upon GIRK1 overexpression (Fig. 3A). Analysis and classification into functionally related groups of genes using the Database for Annotation, Visualization and Integrated Discovery (DAVID) revealed that many of these transcripts are regulated towards specific cellular functions and pro-tumorigenic action. In Fig. 3B, significantly regulated clusters that were of interest are shown (see discussion section for detailed consideration of pathways and the role of individual components in breast cancer). Enrichment scores (ES), p-values and FDRs for all significant clusters are shown in Supplementary Table S3. Heat maps of selected clusters are shown in Fig. 3C, displaying the quantitative effect that underscores the amount of cellular regulation exerted by GIRK1 overexpression (Fig. 3C). Heat maps of all significantly enriched clusters are shown in Supplementary Figures S3, S4.

### GIRK1 overexpression promotes cellular migration

**Figure 4:**
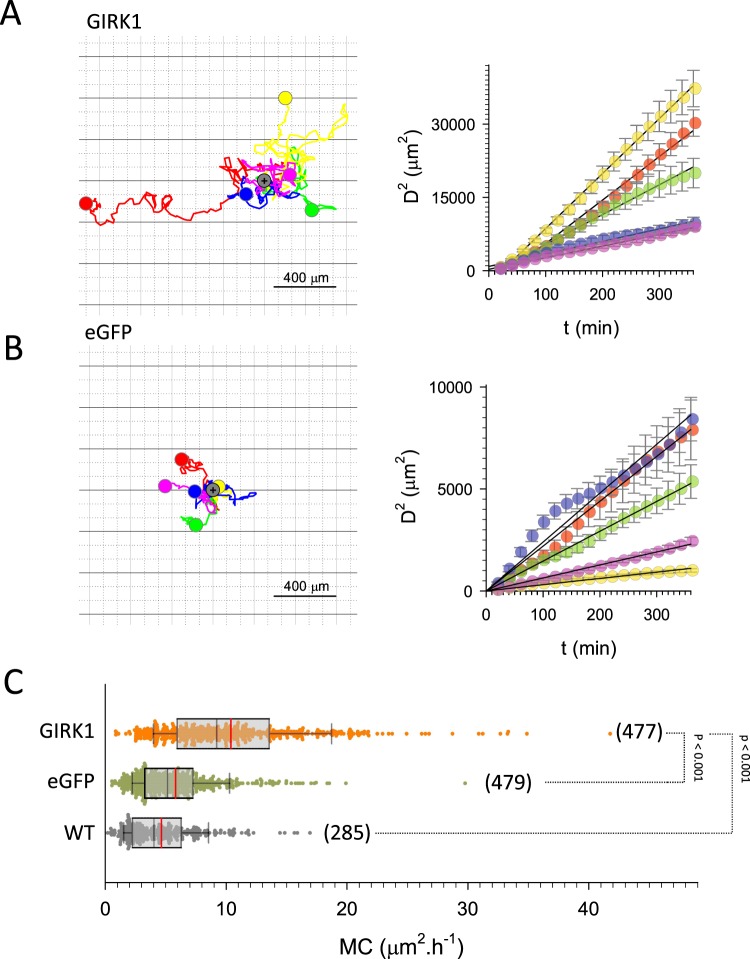
Cellular migration of MCF10A cells. (**A**) Migration of 5 selected MCF10A^GIRK1^ cells over the entire observation interval. *Left:* flower plots showing cellular trajectories. Starting position of each individual cell was set to the same position, indicated by grey circle. Colored circle indicates the positon of a cell after 72 h. *Right:* squared distance as a function of time for the five cells shown to the left (circles; bars indicate standard error). Lines represent linear fits through the data. (**B**) Same as (**A**), but MCF10A^eGFP^. (**C**) Statistical analysis of motility coefficients derived from the different experimental groups. *WT:* MCF10A^WT^, *eGFP:* MCF10A^eGFP^ and *GIRK1:* MCF10A^GIRK1^. The median value is represented by the black line within the box, box margins represent 75% and 25% percentiles, whiskers indicate 90% and 10% percentiles. The red line represents the mean value. Individual values are shown as dots. The number of individual cells is given in parenthesis besides each box. Statistically significant differences between groups are indicated by brackets.

**Figure 5:**
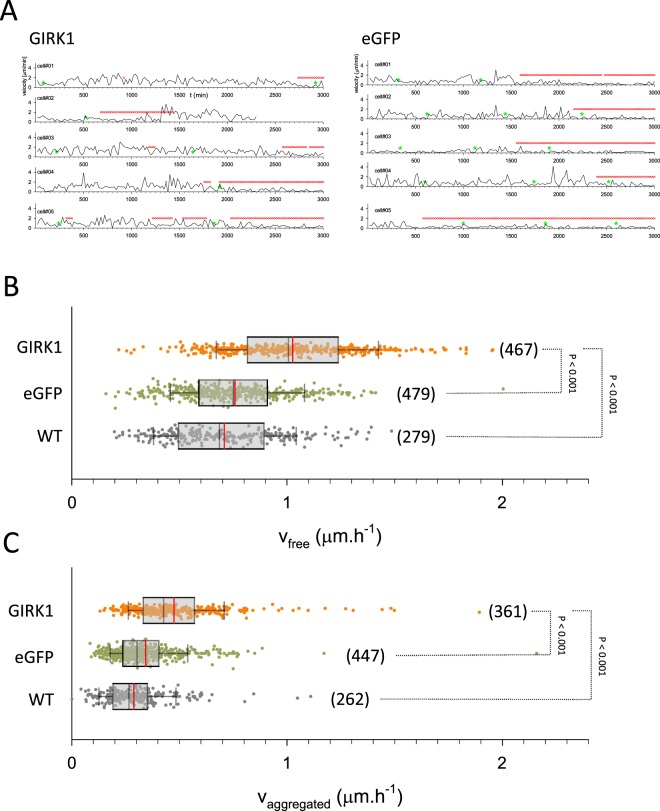
Cellular velocities of MCF10A cells. (**A**) Cellular velocities for five representative cells during the entire observation interval. *Left:* MCF10A^GIRK1^, *right:* MCF10A^GFP^. Red x denotes times were the cell was aggregated, green asterisk indicates cell division. (**B**) Statistical analysis of average cellular velocities of free moving cells derived from the different experimental groups. *WT:* MCF10A^WT^, *eGFP:* MCF10A^eGFP^ and *GIRK1:* MCF10A^GIRK1^. The median value is represented by the black line within the box, box margins represent 75% and 25% percentiles, whiskers indicate 90% and 10% percentiles. The red line represents the mean value. Individual values are shown as dots. The number of individual cells is given in parenthesis besides each box. Statistically significant differences between groups are indicated by brackets. (**C**) Similar to (**B**) but cellular velocities of aggregated cells.

**Figure 6:**
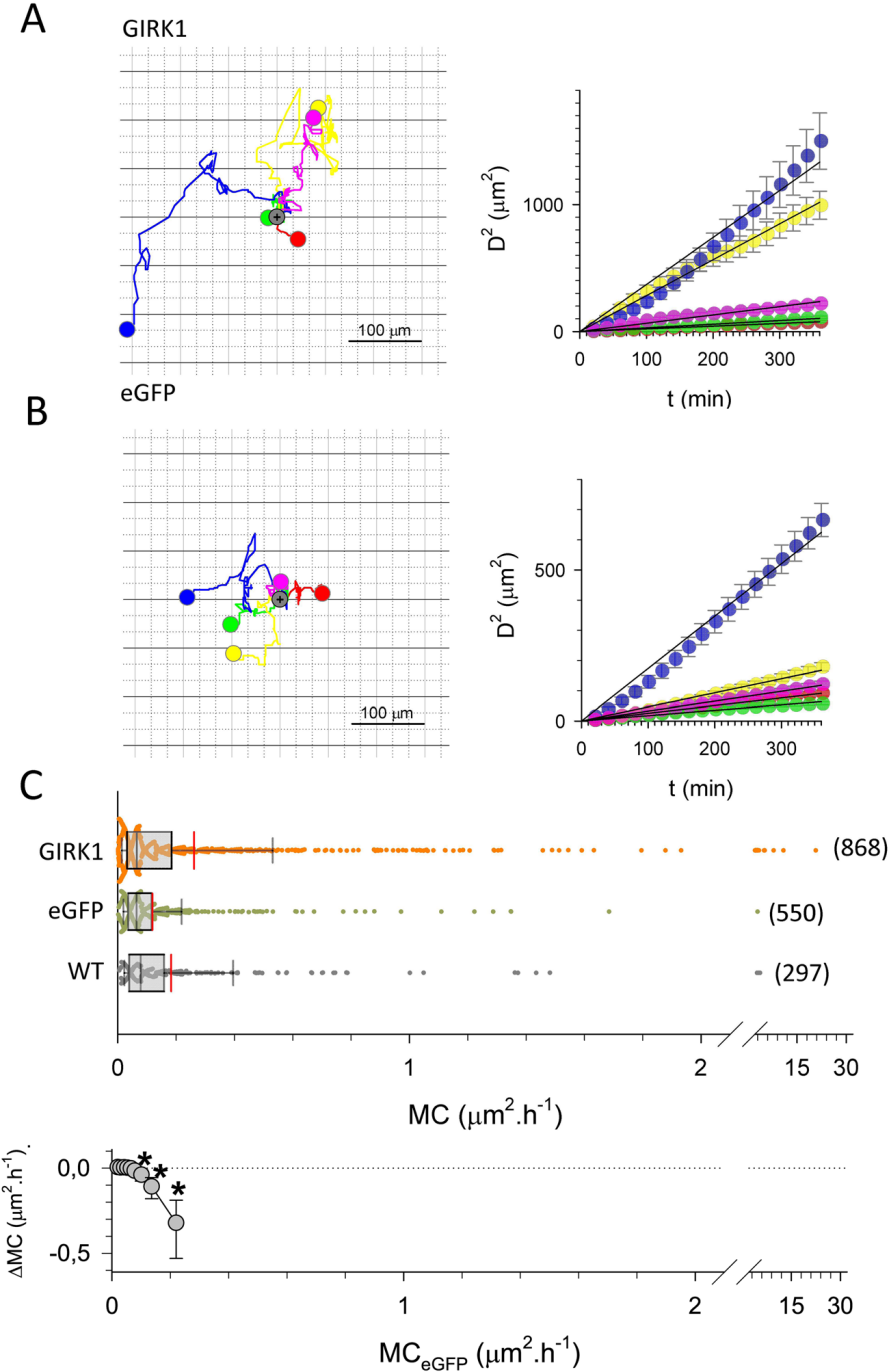
Cellular migration of MCF7 cells. (**A**) Migration of 5 selected MCF7^GIRK1^ cells over the entire observation interval. *Left:* flower plots showing cellular trajectories. Starting position of each individual cell was set to the same position, indicated by grey circle. Colored circle indicates the positon of a cell after 72 h. *Right:* squared distance as a function of time for the five cells shown to the left (circles; bars indicate standard error). Lines represent linear fits through the data.(**B**) Same as (A), but MCF7^eGFP^. (**C**) Statistical analysis of motility coefficients derived from the different experimental groups. *Top: WT:* MCF7^WT^, *eGFP:* MCF7^eGFP^ and *GIRK1:* MCF7^GIRK1^. The median value is represented by the black line within the box, box margins represent 75% and 25% percentiles, whiskers indicate 90% and 10% percentiles. The red line represents the mean value. Individual values are shown as dots. The number of individual cells is given in parenthesis besides each box. *Bottom:* Difference in MCs (ΔMC = MC_eGFP_-MC_GIRK1_) calculated for 10% percentile intervals vs. MC_eGFP_. Whiskers represent 95% confidence intervals. *The average MCs of the 70%, 80% and 90% percentile differs statistically significant between MCF7^eGFP^ and MCF7^GIRK1^.

**Figure 7:**
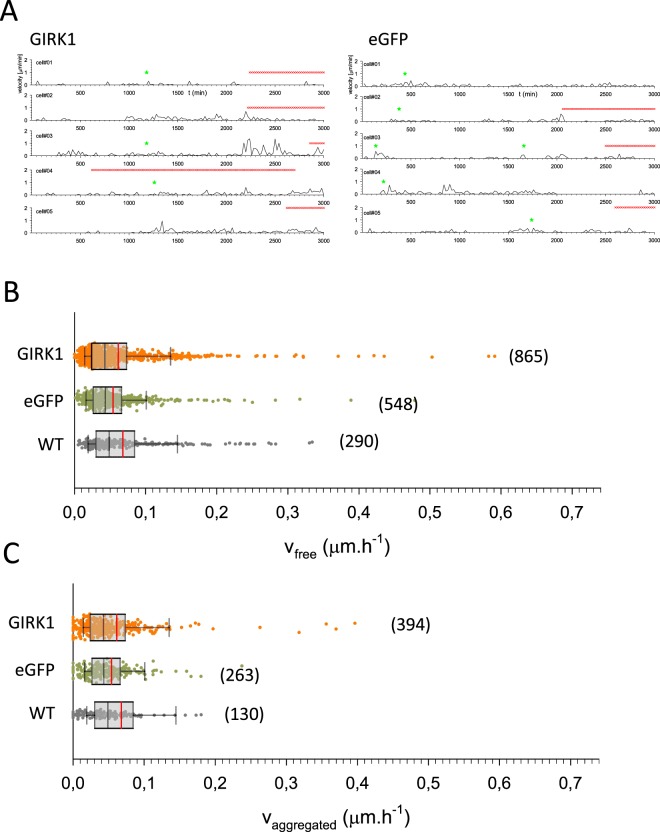
Cellular velocities of MCF7 cells. (**A**) Cellular velocities for five representative cells during the entire observation interval. *Left:* MCF7^GIRK1^, *right:* MCF7^GFP^. Red x denotes times were the cell was aggregated, green asterisk indicates cell division. (**B**) Statistical analysis of average cellular velocities of free moving cells derived from the different experimental groups. *WT:* MCF7^WT^, *eGFP:* MCF7^GFP^ and *GIRK1:* MCF7^GIRK1^. The median value is represented by the black line within the box, box margins represent 75% and 25% percentiles, whiskers indicate 90% and 10% percentiles. The red line represents the mean value. Individual values are shown as dots. The number of individual cells is given in parenthesis besides each box. There was no statistically significant difference between the individual groups. (**C**) Similar to (**B**) but cellular velocities of aggregated cells.

GIRK1 overexpression in MCF10A triggered the downregulation of GO clusters about cell migration, motility, and locomotion (In particular GO:0006928, GO:0030335, GO:2000147, GO:0051272, GO:0040017, GO:0040011, GO:0030334, GO:2000145, GO:0040012, GO:0016477, GO:0051270, GO:0051674, GO:0048870, GO:0006935 and GO:0042330; see also Supplementary Table 3). Many genes in these GO terms promote cellular migration and metastatic spread of tumor cells (see discussion section for selected examples). The fact that GIRK1 overexpression leads to downregulation of these GO terms and genes prompts to study cellular motility and velocity of the MCF10A and MCF7 based cell lines. GIRK1 overexpression greatly enhanced migration of MCF10A as assessed via cellular motility coefficient (Fig. 4; see supplementary videos for representative examples of each experimental group (MCF10A_GIRK1_motility.mp4; MCF10A_eGFP_motility.mp4; MCF10A_WT_motility.mp4; MCF7_GIRK1_motility.mp4; MCF7_eGFP_motility.mp4 and MCF7_WT_motility.mp4)). Accordingly, cellular velocities were substantially increased (Fig. 5). Enhanced migration could also be observed in malignant MCF7^GIRK1^ cells, but the effect was muted compared to MCF10A. The most motile third of MCF7^GIRK1^ cells displayed increased cell motility when compared to MCF7^eGFP^, while cellular velocities were virtually unchanged (Figs. 6, 7).

### GIRK1 overexpression defers wound healing in benign MECs

**Figure 8:**
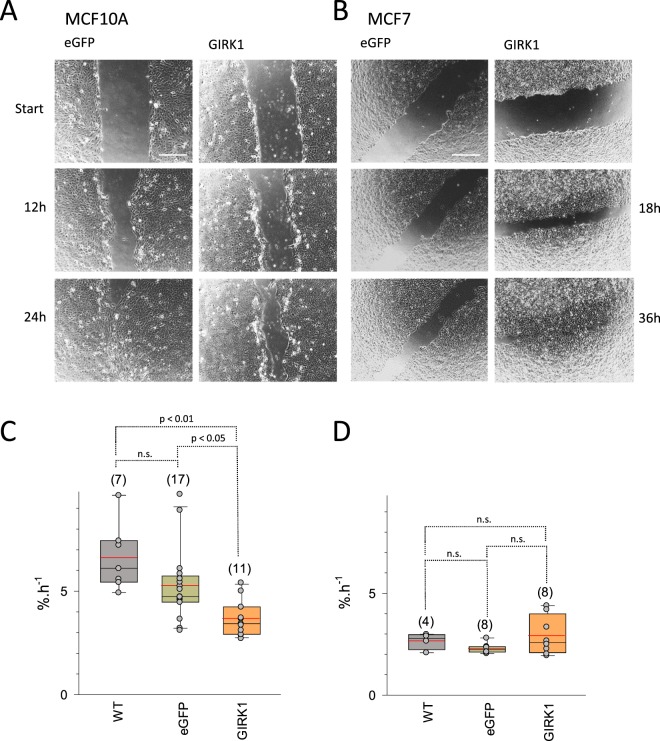
Wound healing rates in the different MEC lines. (**A**) Representative frames recorded at different time points during the wound healing process. *eGFP:* MCF10A^eGFP^ and *GIRK1:* MCF10A^GIRK1^. (**B**) Similar to (A), but MCF7. (**C**) Statistical analysis of wound healing rates of MCF10A cells. *WT:* MCF10A^WT^, *eGFP:* MCF10A^eGFP^ and *GIRK1:* MCF10A^GIRK1^. The median value is represented by the black line within the box, box margins represent 75% and 25% percentiles, whiskers indicate 90% and 10% percentiles. The red line represents the mean value. Individual values are shown as grey circles. The number of individual experiments is given in parenthesis above each box. Statistically significant differences between groups are indicated above brackets (n.s.: statistically not significant). (**D**) Same as (**C**), but MCF7 cells.

Collective cell migration in adulthood takes place exclusively during tissue repair, e.g. when collective migration of epidermal sheets occurs across the provisional wound-bed, leading to epidermal wound closure. Similar to formative collective movements during growth and regeneration in the sound body, collective movement also occurs in cancer but aggravates the course of disease^21^. Wound healing rates of MCF10A^GIRK1^ cells were substantially reduced when compared to controls (MCF10A^WT^ and MCF10A^GFP^; Fig. 8; see supplementary videos for representative examples of each experimental group (MCF10A_GIRK1_WH.mp4; MCF10A_eGFP_WH.mp4; MCF10A_WT_WH.mp4; MCF7_GIRK1_WH.mp4; MCF7_eGFP_WH.mp4 and MCF7_WT_WH.mp4)). In contrast, overexpression of GIRK1 led to a small increase of the wound healing rate in MCF7 cells (this increase was not statistically significant). In summary, our results demonstrate that GIRK1 expression has a distinct influence on wound healing in the malign and benign MEC lines.

### Extracellular matrix interactions of benign MECs are hampered by GIRK1 overexpression

**Figure 9:**
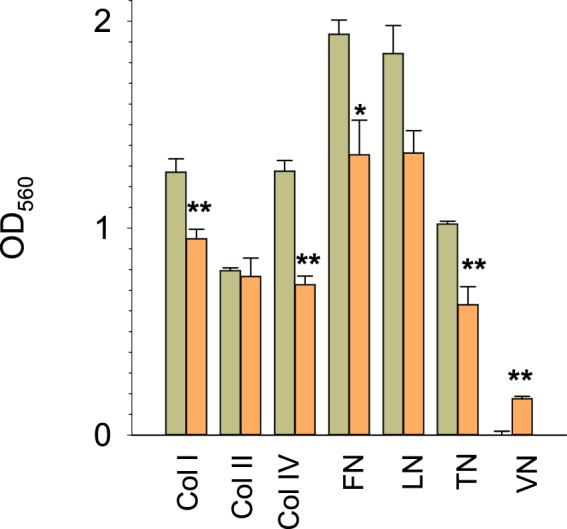
Interaction of MCF10A with different ECM components. Cell adhesion (OD_560_ values) of MCF10A^eGFP^ (*green*) and MCF10A^GIRK1^ (*orange*) to surface coated with different extracellular matrix molecules: *Col I:* human Collagen Type I, *Col II:* human Collagen Type II, *Col IV:* human Collagen Type IV, *FN:* human Fibronectin, *LN:* human Laminin, *TN:* human Tenascin-C and *VN:* human Vitronectin. Mean values ± SEM for three or four experiments for each experimental group are shown. *(**): The mean values differ statistically significant at the p < 0.05 (p < 0.001) level.

MCF10A^GIRK1^ differed markedly to MCF10A^eGFP^ regarding surface adherence properties as revealed by adhesion to surface coated with different extracellular matrix (ECM) molecules (Fig. 9). Of note, the most prominently transcriptionally deregulated gene cluster in MCF10A^GIRK1^/MCF10A^eGFP^ is predicted to weaken interaction of cells with ECM molecules upon downregulation. Collagen I, collagen IV, fibronectin and tenascin-C adherence were hampered by GIRK1 overexpression. The interaction with laminin-coated surface was also weaker, but statistically not significant. Only cell adhesion to vitronectin coated surface, which was virtually undetectable in the control MCF10A^eGFP^ line, was marginally increased in MCF10A^GIRK1^.

### Proliferation of MEC in culture remains unchanged upon GIRK1 overexpression

**Figure 10:**
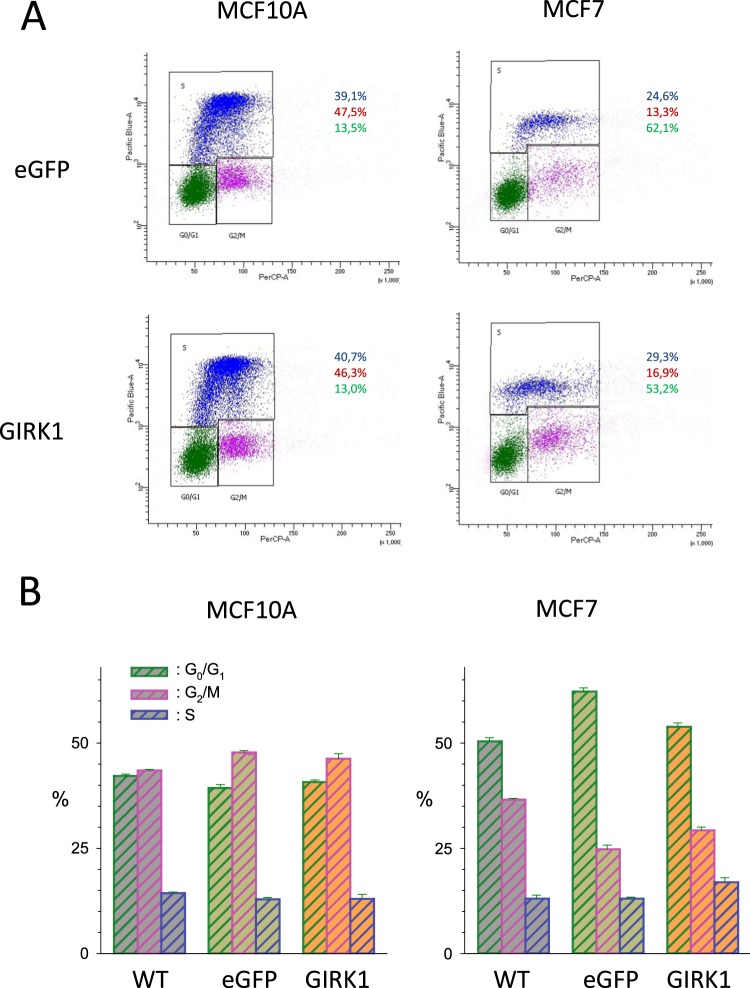
Proliferation of MEC lines in cell culture. (**A**) Representative original results from cell cycle assessment by gated cell sorting according to fluorescence intensities (x-axis: PerCP-A; y-axis: PacificBlue). *Left:* MCF10A^eGFP^ (*top*) and MCF10^GIRK1^ (*bottom*). *Right:* MCF7^eGFP^ (*top*) and MCF7^GIRK1^ (*bottom*). Individual cells (represented as dots), attributed to a particular phase of the cell cycle are surrounded by black line (*green dots:* G_0_/G_1_ phase; *purple dots:* G_2_/M phase and *blue dots:* S phase). (**B**) Statistical analysis of the percentage of time spend at different stages of the cell cycle. *Left:* MCF10A^WT^, MCF10A^eGFP^ and MCF10A^GIRK1^. *Right:* MCF7^WT^, MCF7^eGFP^ and MCF7^GIRK1^. The differences between WT, eGFP and GIRK1 do not differ statistically significant for either MCF10A nor MCF7.

Transcriptome analysis revealed profound downregulation of several gene clusters that predict augmentation of cell division, chromosome segregation and cellular proliferation. This led us to expect a strong impact of GIRK1 overexpression on cellular proliferation. In contrast to our expectations, proliferation of MECs in culture was virtually unaffected upon GIRK1 overexpression, both in MCF10A as well as in MCF7 cells (Fig. 10). Similarly, colony formation assay did not reveal any change between MEC lines in their ability to form colonies, nor in the growth rate of the colonies formed (Supplementary Figure S2).

### GIRK1 overexpression in MCF10A does not interfere with neovascularisation

**Figure 11:**
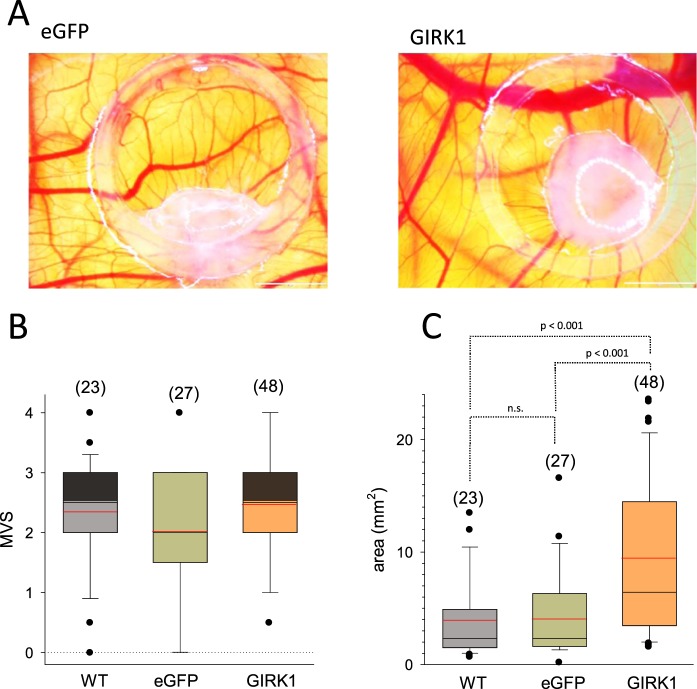
Neovascularization and growth of MCF10A lines planted on chicken embryo. (**A**) Representative image of onplant taken on day 4 after beginning. *Left:* MCF10A^eGFP^ cells. *Right:* MCF10A^GIRK1^ cells. White scalebar is 2 mm. (**B**) Neovascularization of MCF10A lines as assessed by macroscopic vascularization score (MVS). *WT*: MCF10A^**WT**^, *eGFP*: MCF10A^eGFP^ and *GIRK1*: MCF10A^GIRK1^. Box plots represent median value, 25% and 75% percentiles, respectively. Mean values are indicated by red line. Whiskers denote 10% and 90% percentiles. Single scores outside the 10% and 90% percentiles are depicted as black circles. Number of individual onplants is given in parenthesis above each box. Differences were not significant at the statistical level. (**C**) Onplant area after 4 days of growth. *WT*: MCF10A^**WT**^, *eGFP*: MCF10A^eGFP^ and *GIRK1*: MCF10A^GIRK1^. Box plots represent median value, 25% and 75% percentiles, respectively. Mean values are indicated by red line. Whiskers denote 10% and 90% percentiles. Black circles denote single values outside the 10% and 90% percentiles. Number of individual onplants is given in parenthesis above each box. P-values for statistically significant differences are indicated above brackets (n.s.: statistically not significant).

To address the neovascularization potential of MCF10A^GIRK1^, the chick chorioallantoic membrane (CAM) assay was performed. Neovascularization of MCF10A^GIRK1^ onplants by the host equaled controls (MCF10A^eGFP^ & MCF10A^WT^; Fig. 11A,B). Accordingly, gene clusters responsible for neovascularization were unchanged in MCF10A^GIRK1^ when compared to MCF10A^eGFP^. Also in malignant MECs, GIRK1 overexpression turned out to be ineffective for neovascularization, when MCF7^GIRK1/eYFP^ cells displayed vascularization scores close to control^18^. GIRK1 overexpression in MCF10A^GIRK1^ induced, however, a massive spread of onplant area, when compared to controls (MCF10A^eGFP^ & MCF10A^WT^; Fig. 11C), being in line with the observed reduced cell adhesion induced by GIRK1 overexpression.

## Discussion

The present study demonstrates that overexpression of GIRK1 in benign MECs triggers a profound transcriptional response, which includes numerous genes involved in several cellular pro-tumorigenic pathways. In addition, GIRK1 overexpression in MCF10A results in tertiapin-Q sensitive hyperpolarization of resting potential, indicating that GIRK1 overexpression leads to the formation of functional transmembrane GIRK channels that are already active to some basic extent, even in the absence of agonists. Agonist independent, basal, activity of GIRK channels indicates the abundance of endogenous free Gβ/γ dimers^22^. Since additional GIRK isoforms, other than GIRK1, are thought to be indispensable for functional heterotetrameric K^+^ channel formation^1^, the question arises whether GIRK1 isoforms are present in MECs. GIRK4 protein pre-exists in benign MECs, including MCF10A^17^, while GIRK4 and GIRK2 have been identified in the malign MCF7 line^16^. In the malign MCF7 line, GIRK1 overexpression resulted in hyperpolarized membrane resting potential. The effect was less pronounced in comparison to MCF10A. Blocking of GIRK channels in MCF7^GIRK1^ by tertiapin-Q results in even stronger depolarization compared to the MCF7 control groups. We attribute the value of resting membrane potentials in the controls to moderate overexpression of GIRK1 that is already preexisting in the malignant MCF7 line^17^. Similar to the observed effect on the membrane resting potential, silencing of preexisting GIRK complexes in MCF7 by overexpression of the dominant negative splice variant GIRK1d, had a bearing on several vital functions of MCF7 cells which were in opposition to the ones of the functional GIRK1a variant^18^. Thus, preexisting GIRK complexes already act electrogenic and exert regulatory actions on cellular properties of the malignant MCF7 cell line. The effects induced by GIRKs are further increased upon ectopic GIRK1 overexpression in MCF7. Upon GIRK1 overexpression, the motility and cellular velocities of the benign MCF10A control cell lines were substantially increased and associated with transcriptional changes of genes that are linked to motility at the cellular level as shown in the heat map “Cell migration” (Fig. 3C). Apart from their effect on cellular motility, the following genes are important in mammary carcinogenesis and breast cancer progression: IL-1ß is pivotal for pathogenesis and is understood to contribute to the poor prognosis of estrogen-dependent breast cancers^23^. Homozygous deletions of DOCK8 exist in breast cancer and lung cancer^24^. Reduced CCBE1 expression has shown to result in increase of invasive capacity in breast cancer cells and to lead to poor clinical prognosis in patients^25^. In benign MECs, Notch-1 activation triggers downregulation of LAMC2 resulting in cellular responses towards cancer hallmarks, including cell migration^26^. MTSS1 downregulation causes early carcinogenesis and results in increased metastatic spread as well as poor survival rates in breast cancer and lung cancer^27,28^. In comparison to the benign MCF10A cell line, the impact of GIRK1 overexpression on motility of the malign MCF7 cell line was less pronounced. Nevertheless, these findings corroborate our previous observation of increased migration of MCF7^GIRK1/eYFP^ compared to controls^18^. Upon GIRK1 overexpression, wound healing was highly impaired in the benign MCF10A line, with little influence on the malignant MCF7 MEC line. Similarly, Rezania *et al*. observed a small increase in wound healing rate upon overexpression of GIRK1/eYFP chimeric protein in the malignant MCF7 line, but a decrease when GIRK1 function was knocked out through overexpression of the dominant negative splice variant GIRK1d^18^. Hence, marginal GIRK1 levels, as observed in MCF7^WT^, have negative bearing on wound healing. Impairment of wound healing is considered a cancer hallmark^29^ and is co-opted as element of the developmental regulatory program, referred to as the “epithelial-mesenchymal transition” (EMT), by which transformed epithelial cells are able to acquire the abilities to invade, to resist apoptosis, and to disseminate^30^. Transcriptional downregulation of the gene cluster “wound healing” was revealed by transcriptome analysis (see heat map “wound healing”; Fig. 3C). Among the genes, downregulated in MCF10A^GIRK1^ and linked to impairment of wound healing, were DOCK8 and CD36 that are relevant players in breast cancer^24,31^. Breast cancer is often associated with massive alterations of ECM architecture and composition, influencing a number of biological activities such as cell proliferation, differentiation, biosynthetic ability, polarity, locomotion and mechanical stress via structurally different surface receptors^32^. GIRK1 protein possesses an extracellular integrin binding motif^33^ and hence may contribute to cell-ECM interactions. In the present study, overexpression of GIRK1 in MCF10A led to reduced adherence to surfaces coated with various extracellular matrix molecules. These experimental observations were reflected by downregulation of genes comprised in a functional cluster termed “Extracellular matrix – receptor interaction” (Fig. 3C). Among the identified transcripts, downregulation of COL6A2, SERPINA1, CCBE1, TFPI2, THBS1 and COL8A1 have been shown to promote migration and invasion of malign breast cancer cells, aggravate metastatic spread and result in poor prognosis of breast cancer patients^25,34–37^. Based on preclinical experiments, using the malignant MCF7 line, Chen *et al*.^35^ suggested that low SERPINA1 expression levels may be a strong indicator for bad response of ER^+^ and ER^+^/HER2^+^ breast tumors to anti-ER as well as anti-HER2 therapies. Both the MCF10A as well as the MCF7 cell lines did not display alterations in proliferation, ability to form colonies upon seeding on petri dishes nor in growth rates of the colonies formed. Rezania *et al*.^18^ obtained similar results upon overexpression of chimeric GIRK1/eYP protein in the malignant MCF7 cell line. The lack of effect of GIRK1 overexpression on cell proliferation is, to some extent, unexpected since GIRK1 overexpression in MCF10A has induced resilient changes in several gene clusters involved in cell division and proliferation. We hypothesize that the alterations in gene expression observed may require intrinsic factors from e.g. the tumor microenvironment, that are missing in our 2D cell culture, to become manifest in the functional phenotype. The gene clusters discussed above exert protumorigenic action upon downregulation. The most prominently regulated functional cluster is, however, upregulated and comprises downstream elements which are triggered by the cytokine interferon-γ (IFγ; “Interferon-γ response” (Fig. 3C)). Canonical IFγ signaling causes activation of transmembrane IFγ receptors (IFγR1 and IFγR2), subsequent activation of JAK tyrosine kinases, binding and nuclear translocation of STAT proteins and subsequent activation of transcription factors such as interferon response factors (IRFs^38^). Under physiological settings, IFγ signaling brings about biological functions related to antibacterial and antiviral host defense and immune regulation, cell cycle, apoptosis, inflammation and innate and acquired immunity^38^. The resilient immunomodulatory functions implicated in IFγ signaling prompted several clinical applications for the cure of chronic granulomatous disease, fungal infections, autoimmune diseases and cancer^39^. At about the same time that IFγ was regarded a promising antitumor agent, IFγ had been identified to exert another, vicious, side and to promote metastatic spread, resistance against natural killer cells, anti-apoptotic and proliferative responses of various malignant cell types (see^40,41^, for review) and, in the pathophysiological context of malignant cells and microenvironment, acts pro-tumorigenic^42^. When screening radiation- and chemotherapy-resistant tumors, Khodarev *et al*.^43^ and Weichselbaum *et al*.^44^ discovered a gene signature, termed interferon-related DNA damage resistance signature (IRDS), because the majority of the genes identified were stimulated by interferon and are activated during antiviral response. In breast cancer, IRDS genes are regulated by exosome transfer from stroma cells, mediate resistance to radiation- and chemo-therapy, predict poor prognosis and are enriched in cells exerting cancer stem cell like features^45^. In the present study, overexpression of GIRK1 in the benign MCF10A line triggered enhanced expression of several IRDS genes such as IFI6, IFI27, IFI44, IFIT1, IFIT3, IRF7 and ISG15, indicating a shift towards this awkward signature. Among the most strongly upregulated downstream IFγ signaling elements are IFI6, DCLK1 and LCN2, which have been shown to promote migration, invasion and metastasis formation of malignant MECs and to correlate with poor prognosis for patients (^46–48^). Likewise, the type 1 interferon/IRF7 axis has been shown to bring about chemotherapy-induced immunological dormancy in breast cancer^49^.

This is the first study to provide insight into the cellular and molecular consequences of GIRK1 overexpression in benign MCF10A cells. Our study reveals that the overexpression of a single K^+^ channel subunit shifts vital parameters of a benign MEC line towards cancer hallmarks. Expression levels of a sizeable number of transcripts, several of them involved in induction, progression and resistance to the therapy of treatment of breast cancer, accompanies the changes in cellular behavior. The observed influence on cellular behavior goes well beyond the role of overexpression of a K^+^ ionophore acting on cellular motility via topical osmoregulatory K^+^ levels alone. It indicates that GIRK1 may represent a missing link for the complementation of pathophysiological de-novo pathways that alter cellular homeostasis towards the malign phenotype. This is in accordance with the observation of excessive GIRK1 mRNA and protein levels in breast tumors that correlate with poor prognosis for patients with ER^+^ breast cancer^14^. Hence, the future and detailed investigation of GIRK1 mediated pathways in MECs is likely to provide essential insight into development and progression especially in the subset of ER^+^ breast cancer. Through its pronounced influence on intracellular signaling cascades, GIRK1 overexpression may open a new window for treatment and classification of breast tumors.

## Materials and Methods

Commercial providers of chemicals and reagents are listed in Supplementary Table S1. All chemicals were reagent grade, unless stated otherwise.

### Cell culture and media

MCF7 cells were maintained in MEM supplemented with 10% FBS, 1% L-Glutamine, 1% Sodium Pyruvate and Penicillin-Streptomycin (100 U/ml and 100 ng/ml) at 37 °C with 5% CO_2_. MCF10A cells were cultured in MEBM medium including SingleQuots and 0,004% CT. WT cell lines were used between passages P27 to P35 and split every 3 to 5 days. Stably transfected cell lines were used since generation as follows: MCF7^eGFP^: P8-P13; MCF7^GIRK1^: P10-P16; MCF10A^eGFP^: P7-P13; MCF7^GIRK1^: P11-P16. All cell lines were free of mycoplasma contamination, as tested routinely. To prove the common origin of MEC lines, short tandem repeat (STR) profiling of the parental cell lines was performed. All markers (CSF1PO, DSS818, D13S317, D7S820, D16S539, vWA, TH01, TPOX and Amelogenin) indicated integrity of the cell lines.

### Constructs and transfection

For the generation of stable constructs, the coding sequence of human GIRK1 was amplified by PCR (NM_002239.3; primers used: plasmid-GIRK1a-f, plasmid-GIRK1a-r; see Supplementary Table S2 for sequence), digested with NheI and BamHI and cloned into the cloning site of pIRES2eGFP. Sanger sequencing verified the sequence of the entire construct. For stable transfection, the pIRES2eGFP plasmid was linearized with AseI. Stable transfection of MCF10A or MCF7 cells was achieved using Lipofectamine following the manufacturer’s protocol. 24 h after successful transfection, as verified by fluorescence microscopy, appropriate medium supplemented with G418 was added (16 mg/mL for MCF10A and 3 mg/mL for MCF7, respectively). After 5 to 7 days of growth, transfected cells were isolated by cell sorting using eGFP fluorescence as reporter acquired on a LSRII (BD Bioscience, Vienna, Austria). Stable transfection was verified by routinely checking the cells for fluorescence by microscopy throughout the duration of the investigation.

### RNA isolation

Cells were grown to 70–80% confluence, harvested by trypsinization and spun at 750 rpm for 3 minutes. RNA was isolated from cell-pellet using QIA shredder spin column and RNeasy Mini Kit according to manufacturer’s protocol.

### Transcriptome analysis by next-generation sequencing

10 ng of isolated RNA was heat inactivated for 10 min at 80 °C followed by cDNA Synthesis using the SuperScript VILO cDNA synthesis kit. DNA library was generated using the Ion AmpliSeq Transcriptome Human Gene Expression kit according to manufacturers’ instructions. Sequencing was performed on Ion S5 XL benchtop sequencer (Thermo Fisher Scientific) to a length of 200 base pairs. On average 8 million reads were sequenced per sample. Data analysis was performed using the Ampliseq RNA Transcriptome workflow of the Ion Torrent suite. Briefly, amplicons were mapped to a human transcriptome reference (AmpliSeq_Transcriptome_21K.v1) and mapping reads were counted for each transcript and exported to a tabular file. Differentially expressed genes between GIRK1 and eGFP expressing cells were defined through 2-fold difference and a coefficient of variation below 0.7 between the two replicates. DAVID functional clustering was performed with up- and downregulated genes separately, selecting gene ontology (GO) biological processes and KEGG pathways^50,51^. Significant enrichment was determined via false discovery rate (FDR) and/or Benjamini-Hochberg corrected value. Heat maps were generated using Genesis^52^. RNA-seq expression of six genes, selected from the clusters “IFγ”, “ECM” and “migration” was confirmed by independent qPCR (Validation of RNA-seq data is shown in Supplementary Figure S5). Original data have been deposited in NCBI’s Gene Expression Omnibus (Accession number: GSE138155; GEO; https://www.ncbi.nlm.nih.gov/geo/).

### qPCR

For converting to cDNA, QuantiTect Rev the Transcription kit was used according to manufacturer’s protocol. The obtained cDNA was diluted 1:25 and quantitative real-time PCR was performed with the Thermal Cycler C1000 Touch in 96-well plates, using QuantiFast SYBR Green PCR Kit, according to manufacturers’ instructions. All samples were measured in triplicate. The relative mRNA expression levels of GIRK1 gene compared to the housekeeping genes YWHAZ and GAPDH were calculated using the 2^ΔΔCt^ method^53^. GIRK1 mRNA levels were normalized to the one of MCF10A^WT^ (See Supplementary Table S2 for primer sequences).

### Western blot analysis

For preparation of protein lysate from MCF10A and MCF7 cell lines, two TC Dishes 35 mm at 90% confluence were used. Cells were washed twice with ice-cold PBS and lysed in 200 µl Frackelton buffer including complete Mini Protease inhibitor Cocktail tablet. Lysates were sonicated for 30 minutes, using an ultrasound device (ECONO-Clean TVEN), followed by centrifugation at 13200 rpm (4 °C; 15 min). Protein concentration was determined^54^. In each slot 3 µg protein were loaded onto 12% SDS gels^55^. Spectra Multicolor protein ladder was used as standard. After electro transfer (150 mA, on ice; transfer buffer) on Immobilon-P PVDF, protein bands were located by staining with Ponceau S solution (0,5% diluent) according to the manufacturer’s instructions. Membranes were blocked with 3% Difco Skim Milk in TBST (Tris-buffered saline containing Tween 20) for 30 minutes at room temperature and incubated with primary Ab (overnight; 4 °C). The membranes were washed (3×) with 3% Difco Skim Milk in TBST for 10 minutes and incubated with the secondary antibody (1 hour in room temperature). After incubation with ECL-Select antibody–protein complexes were analyzed by ChemiDoc Imager (BioRad, ChemiDoc MP). The following combinations of primary and secondary antibodies were used: #1.: GIRK1^CT^ Ab (APC-005, directed against GIRK1 C-terminus; Alomone labs, Jerusalem, Israel, rabbit polyclonal) dilution 1:800 in 5% [w/v] BSA with Peroxidase Goat anti-rabbit as secondary Ab (1:10000 in 3% [w/v] skim milk); #2.: GIRK1^NT^ Ab (directed against GIRK1 N-terminus; rabbit polyclonal, produced by Kurt Schmidt, dilution 1:250 in 5% [w/v] BSA) with Clean Blot IP detection reagent as secondary Ab (1:5000 in 3% [w/v] skim milk); #3.: monoclonal GIRK1 Ab (Abcam No. 119246) mouse monoclonal (dilution 1:500 in 5% [w/v] BSA) with Peroxidase Goat anti-mouse as secondary Ab (1:10000 in 3% [w/v] skim milk).

### Immunoprecipitation

MCF7 cells in T75 culture flask at 90% confluence were lysed in 500 µl Frackelton buffer as described under methods for WB. After centrifugation, 150 µl Protein A Agarose was added to the supernatant and incubated for 1 hour at 4 °C. The sample was centrifuged at 4000 rpm (3 minutes, 4 °C). 15 µl GIRK1 (Alomone) antibody (see WB) was added to the supernatant and incubated overnight at 4 °C. 225 µl Protein A agarose suspension was added and incubated at room temperature for 2 hours. The probe was centrifuged a 4000 rpm (3 minutes, 4 °C). After washing with 500 µl Frackelton buffer (3 × ), 30 µl 4x Laemmli buffer was added and incubated (95 °C, 5 minutes) followed by centrifugation (3 min 4000 rpm at RT) and WB analysis was performed.

### Cell adhesion assay

The colorimetric CHEMICON ECM Cell Adhesion Array Kit was used according to manufacturer’s instructions. The ECM components are: Human Collagen Type I (Col I), human Collagen Type II (Col II), human Collagen Type IV (Col IV), human Fibronectin (FN), human Laminin (LN), human Tenascin-C (TN) and human Vitronectin (VN). 1, 0–2, 0 × 10^6^ cells were applied per slot in MEBM medium. The incubation time was two hours at 37 °C. After removal of non-attached cells by pipetting, adhering cells were fixed by cell stain solution. Probes were gently washed and incubated for 10 minutes with extraction buffer. The absorbance at 540–570 nm was determined with a microplate reader (CLARIOstar, BMG LABTECH Inc., Cary, NC, USA).

### Wound healing assay

Cells (1,5 × 10^5^/well) were seeded into 24 well plates. After growing to 90% confluency and using a 200 µl pipette tip, a scratch was made across the center of the well, producing an interruption of the cell monolayer, resulting in a scratch, the width of which was approximately 1 mm. Detached and damaged cells were removed by carefully aspirating the medium and rinsing with PBS before the addition of 700 µl fresh MEBM growth medium. The plates were monitored for 24 h (MCF10A derived lines) or 36 h (MCF7 derived lines) in a Cell Observer (Zeiss Axiovert 200 M) at intervals of 20′ (MCF10A) or 30′ (MCF7), respectively. For analyzing the data, ImageJ^56^ software (1.51j8) and a custom developed macro termed ALGov9 was used (see supplementary materials for complete code).

### Chick chorioallantoic membrane assay

Fertilized white Lohmann chicken eggs were obtained from a local hatchery (Schropper GmbH, Gloggnitz, Austria). The egg shell was cracked on day 3 of embryonic development and embryos were placed into sterilized plastic dishes for further incubation for 7 days in a MultiQuip incubator at 37 °C (50% humidity). MCF10A cells were applied in volumes of 15 µL in a 1:1 mixture with Matrigel (10^6^ cells/onplant) within 5 mm silicone rings. Silicone rings were placed on vascular branches of the CAM. After 4 days of incubation the progression of tumor growth was monitored by photo-documentation (Olympus SZX16). The formed tumors were photographed and neovascularization was scored according to^57^. Onplant area was analyzed by IQM^58^.

### Proliferation assay

Cells (2 × 10^5^) were seeded in 6 well plates and incubated in cell culture incubator with MEBM growth medium. 5-ethynyl-2′-deoxyuridine (EdU) was used to directly measure cells in the S-phase^59^. The Click-iT EdU Pacific Blue Flow Cytometry Assay Kit was used according to the manufacturer’s protocol. After reaching 70–80% confluency, a 10 mM solution of EdU of the Click-iT EdU Assay was added to each well. After 3 hours incubation time, cells were harvested and washed with 3 mL 1% BSA in PBS. Cells without staining and treatment were taken for control in all experiments. To define cell cycle (G_1_/G_0_-; S- and G_2_/M-phases), the fluorescent DNA binding dye 7-AAD viability staining solution (PerCP-A) was used. Flow cytometry of 500 µl cell suspension was performed using LSRII (BD Bioscience, Vienna, Austria). Data were analyzed with DIVA software (BD Bioscience).

### Colony formation assay

One hundred single MCF10A cells were seeded in 1000 µl medium per well in six well plates. For MCF7, 200 single cells were seeded. Following 8 days of culture, plates were washed with PBS solution and fixed for 5 minutes with fixing solution (85% Methanol; 15% Acetic acid). After washing with distilled water the plates were incubated for 2 hours with 1000 µl 0,5% crystal violet stain solution per well, washed with distilled water and air dried at room temperature. The plates were scanned with CanonScan 4400 F. Colony sizes were measured using ImageJ software (1.51j8).

### Electrophysiological recording and analysis

MCF10A and MCF7 cells were seeded on 6 × 6 mm coverslips in 24 well plates, cultured as described for more than 24 h before electrophysiological experiments were started. For recording, the coverslips were transferred to a bathing chamber in bathing solution (NaBS) mounted to the stage of an inverted microscope (IM35, Zeiss, Germany). Using whole cell patch clamp method and nystatin perforation as previously described^60^, resting membrane potentials were recorded at RT not longer than 20 minutes after the cells were removed from the incubator. Briefly, nystatin stock solution (NSS) was prepared by dissolving nystatin (N6261, Merck, Darmstadt, Germany) in DMSO (analytical grade, Merck, Darmstadt, Germany) at a concentration of 50 mg/mL, aliquoted and stored at −20 °C until use. Nystatin containing pipette solution (PSN) was prepared by pipetting 200 μL NSS into 10 mL pipette filling solution (PFS; in 15 mL Falcon tube) under sonication using ultrasonic cleaning bath (Emmi-H22, EMAG AG, Mörfelden-Walldorf, Germany). After nystatin addition, PSN was sonicated for one additional minute, stored on ice and filtered (Wahtman Rotrand 0, 2 μm, FP30/0,2-CA-510462200, Fisher Scientific) immediately before use. Patch clamp pipettes were pulled from borosilicate glass capillaries (Assistent Mikro-Haematokrit capillary tubes, Hecht, Fisher Scientific; final resistance was 1–2.5 MΩ). After Gigaseal formation, perforation was monitored by capacitance measurements. After successful perforation, as judged by capacitance >25 pF, resting membrane potential was low pass filtered at 50 Hz and recorded for 20 seconds using Axopatch-200A amplifier (Molecular Devices, USA). Traces were digitized at 1 kHz using the Digidata 1322 A interface (Molecular Devices, USA) using Clampex 9.2 software (Molecular Devices, USA). Membrane resting potential was measured by averaging the entire trace using the Clampfit 10.3 software (Molecular Devices, USA). The composition of solutions used was as follows (concentrations given in parenthesis in mmol/L): NaBS: KCl (2), NaCl (148), MgCl_2_ (4), CaCl_2_ (1) and HEPES (10), buffered with NaOH to pH:7.4. PFS: KCl (20), K^+^/aspartate^−^ (120), NaCl (10), MgCl_2_ (4), EGTA^−^/K^+^ (1) and HEPES, buffered with KOH to pH: 7.4.

### Motility analysis

Cell movement was recorded and analyzed as described previously^61^, with a recording rate of 3 images per hour over a period of 3 days. Analysis of motility coefficients and cellular velocities was limited to the time interval, when movement was not constrained by the developing cell bulk, using median cell displacement as an indicator. Average cellular velocities were calculated separately for cells that were free or aggregated (defined as cells that were in contact with neighboring cells with >50% of their perimeter).

### Statistical analysis

Statistical analysis of data was performed using SPSS embedded in Sigmaplot (SPW 14.0, Systat Software Inc.). In short, datasets consisting of more than two experimental groups were tested for normal distribution and one way or rank based ANOVA was performed as appropriate. Subsequent pairwise comparisons were performed using appropriate algorithms. For the comparison of two experimental groups, student’s t-test was used. Statistics for cell migration was computed with public domain software R, version 3.5.3 and RStudio software version 1.1.463 (RStudio Team (2016), R Core Team 2019). In case of MCF10A cells, means of data were analysed with a bootstrap-t method based ANOVA using the R function t1waybt (Wilcox 2017), followed by post hoc tests using the R function linconbt allowing also a comparison of all pairs of groups. In case of MCF7 cells, due to pronounced skewness, medians were analysed with a percentile bootstrap method using the R function Qanova followed by pairwise median comparisons using a percentile bootstrap method implemented in the R function medpb^62^. Holm method was used for adjustment of p-values due to multiple comparisons. To compare deciles the R function, qcomhfMC^62^ was used combining a percentile bootstrap method with a quantile estimator allowing tied values.

## Supplementary information

Supplementary information1

Supplementary information2

Supplementary information3

Supplementary information4

Supplementary information5

Supplementary information6

Supplementary information7

Supplementary information8

Supplementary information9

Supplementary information10

Supplementary information11

Supplementary information12

Supplementary information13
