# Supplementary material for: GIRK1 triggers multiple cancer-related pathways in the benign mammary epithelial cell line MCF10A
Source: Sci Rep. 2019 Dec 17;9:19277. doi: 10.1038/s41598-019-55683-w (PMC6917815; doi:10.1038/s41598-019-55683-w)
Supplement: Supplementary file 1 — Supplementary information1 [file 41598_2019_55683_MOESM1_ESM.pdf]

Supplementary Figures & Tables

GIRK1 triggers multiple cancer-related pathways in the benign mammary epithelial cell line MCF10A

SCHRATTER G.[1,*], SCHERUEBEL S.[1,*], LANGTHALER S.[2], ESTER K.[3], PELZMANN B.[1,*], GHAFFARI TABRIZI-WIZSY N.[4], REZANIA S[1,*], GORISCHEK A.[1,*], PLATZER D.[1,*], ZORN-PAULY K.[1,*], AHAMMER H.[1], PROKESCH A.[5], STANZER S.[6], DeVANEY T.T.J.[1,*], SCHMIDT K.[7], JAHN S.W.[8], PRASSL R.[1,*], BAUERNHOFER T.[6,*], SCHREIBMAYER W.[1,*,+].

[1]: Gottfried Schatz Research Center for Cell Signaling, Metabolism and Aging, Medical University of Graz, Austria.

[2]: Institute for Health Care Engineering with European Testing Center of Medical Devices, Technical University of Graz, Austria

[3]: Laboratory of Experimental Therapy, Division of Molecular Medicine, Ruđer Bošković Institute, Bijenička 54, 10000 Zagreb, Croatia

[4]: Institute of Immunology and Pathophysiology, SFL Chicken CAM Laboratory, Medical University of Graz, Austria

[5]: Cell Biology, Histology and Embryology, Medical University of Graz, Austria

[6]: Division of Oncology, Department of Internal Medicine, Medical University of Graz, Austria

[7]: Institute of Pharmaceutical Sciences, Karl Franzens University of Graz, Austria

[8]: Diagnostic & Research Institute of Pathology, Medical University of Graz, Austria

[*]: Research Unit on Ion Channels and Cancer Biology, Medical University of Graz, Austria

[+]: corresponding author: Gottfried Schatz Research Center for Cell Signaling, Metabolism and Aging, Research Unit "Ion Channels and Cancer Biology", Medical University of Graz, Austria, Phone: +43 316 388 71533, Fax: +43 316 385 79611.

Supplementary Figure S1: ***GIRK1 mRNA levels of chimeric MCF7$^{GIRK1/eYFP}$ compared to bicistronic MCF7^{GIRK1} cell lines***

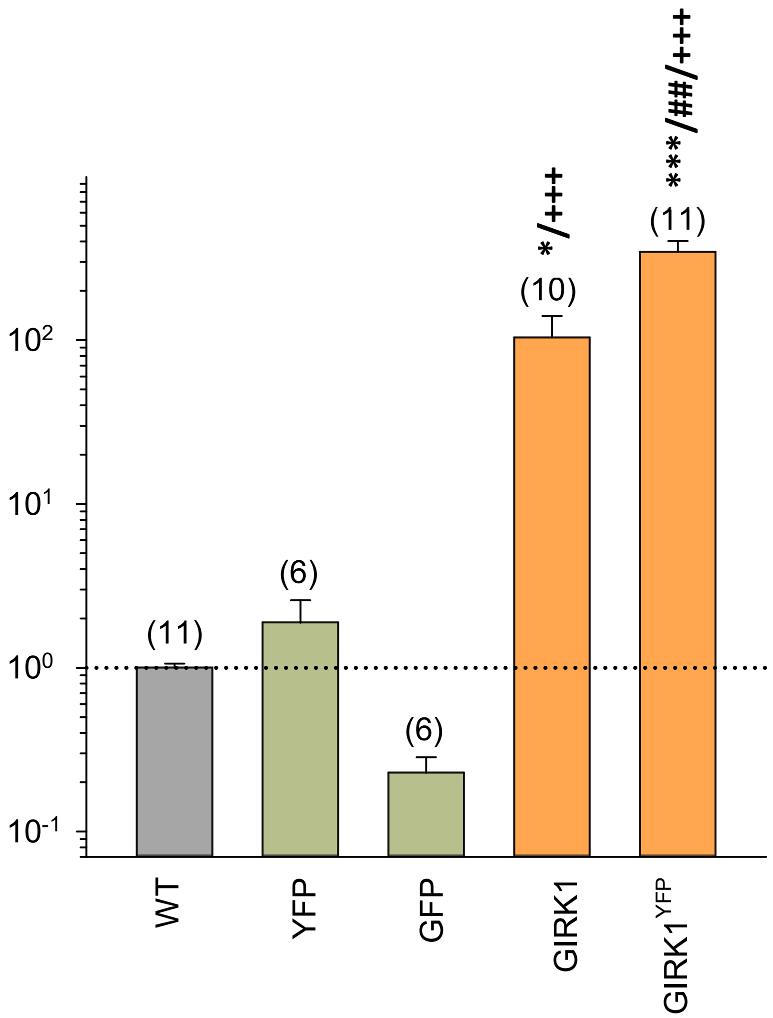

GIRK1 mRNA levels are normalized to MCF7$^{\mathbf{WT}}$. *WT:* MCF7^{WT}, *eYFP:* MCF7^{eYFP}, *eGFP:* MCF7^{eGFP}, *GIRK1:* MCF7^{GIRK1} and *GIRK1^{eYFP}:* MCF7$^{GIRK1/eYFP}$. MCF7^{eGFP} and MCF7^{GIRK1} represent the cell lines engineered using the bicistronic pIRES2eGFP vector described here, while MCF7^{eYFP} and MCF7$^{GIRK1/eYFP}$ represent the chimeric GIRK1/eYFP protein. Number of experiments is given in parenthesis above each bar. *,(***): the group differs statistically significant from MCF7^{WT} $p<0.05$ (<0.001) level. ##: the group differs statistically significant from MCF7^{eYFP}at the $p<0.05$ level. +++: the group differs statistically significant from MCF7^{eGFP} at the $p<0.001$ level.

Supplementary Figure S2: Colony formation and growth assay performed on the different MEC lines

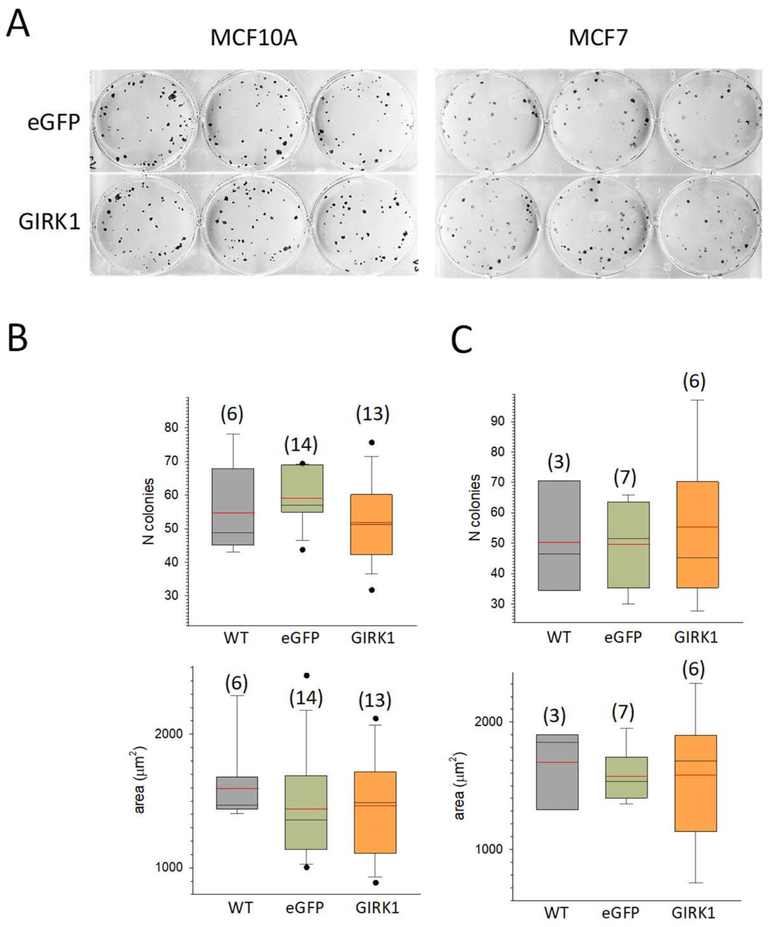

(A) Representative micrographs of three microplate slots per experimental group (stained colonies are visible as dark spots). Diameter of a slot is 35 mm. *Left:* MCF10A^{eGFP} (*top*) and MCF10A^{GIRK1} (*bottom*). *Right:* MCF7^{eGFP} (*top*) and MCF7^{GIRK1} (*bottom*).

(B) Statistical analysis of MCF10A lines. *Top:* Number of colonies formed (per 100 cells seeded out). *WT:* MCF10A^{WT}, *eGFP:* MCF10A^{eYFP} and *GIRK1:* MCF10A^{GIRK1}. *Bottom:* areas of colonies at time of harvesting. *WT:* MCF10A^{WT}, *eGFP:* MCF10A^{eYFP} and *GIRK1:* MCF10A^{GIRK1}. The median value is represented by the black line within the box, box margins represent 75% and 25% percentiles, whiskers indicate 90% and 10% percentiles. The red line represents the mean value. Individual values outside the 10% and 90% percentiles are shown as black circles. The numers of replicates performes is given above each box in parenthesis. The differences between the experimental groups, neither for the number of colonies formed, nor for colony area, are not statistically significant different.

(C) Similar to (B), but results of MCF7 lines are shown. The differences between the experimental groups, neither for the number of colonies formed, nor for colony area, are not statistically significant different.

Supplementary Figure S3: Heat maps of significantly UP-regulated gene clusters:

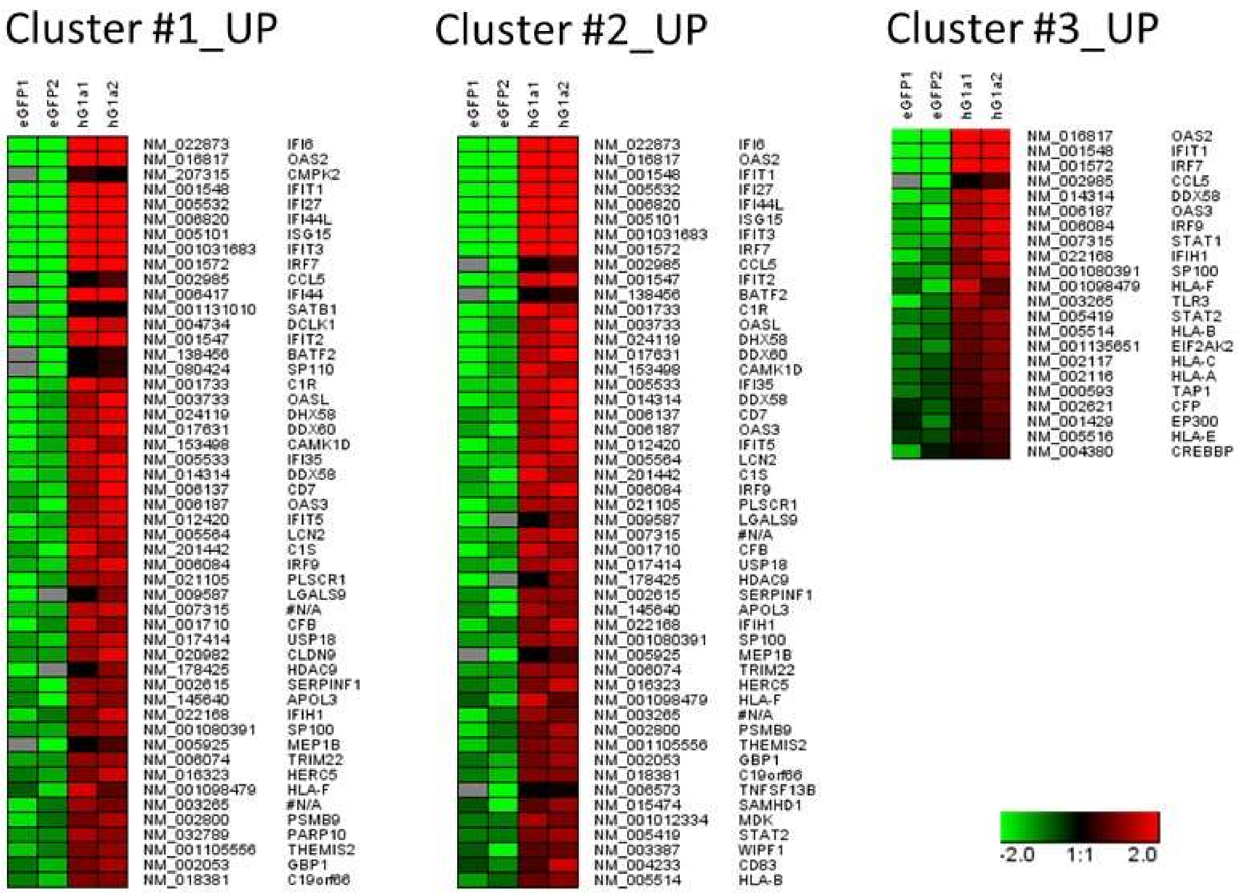

Supplementary Figure S4A: Heat maps of significantly DOWN-regulated gene clusters:

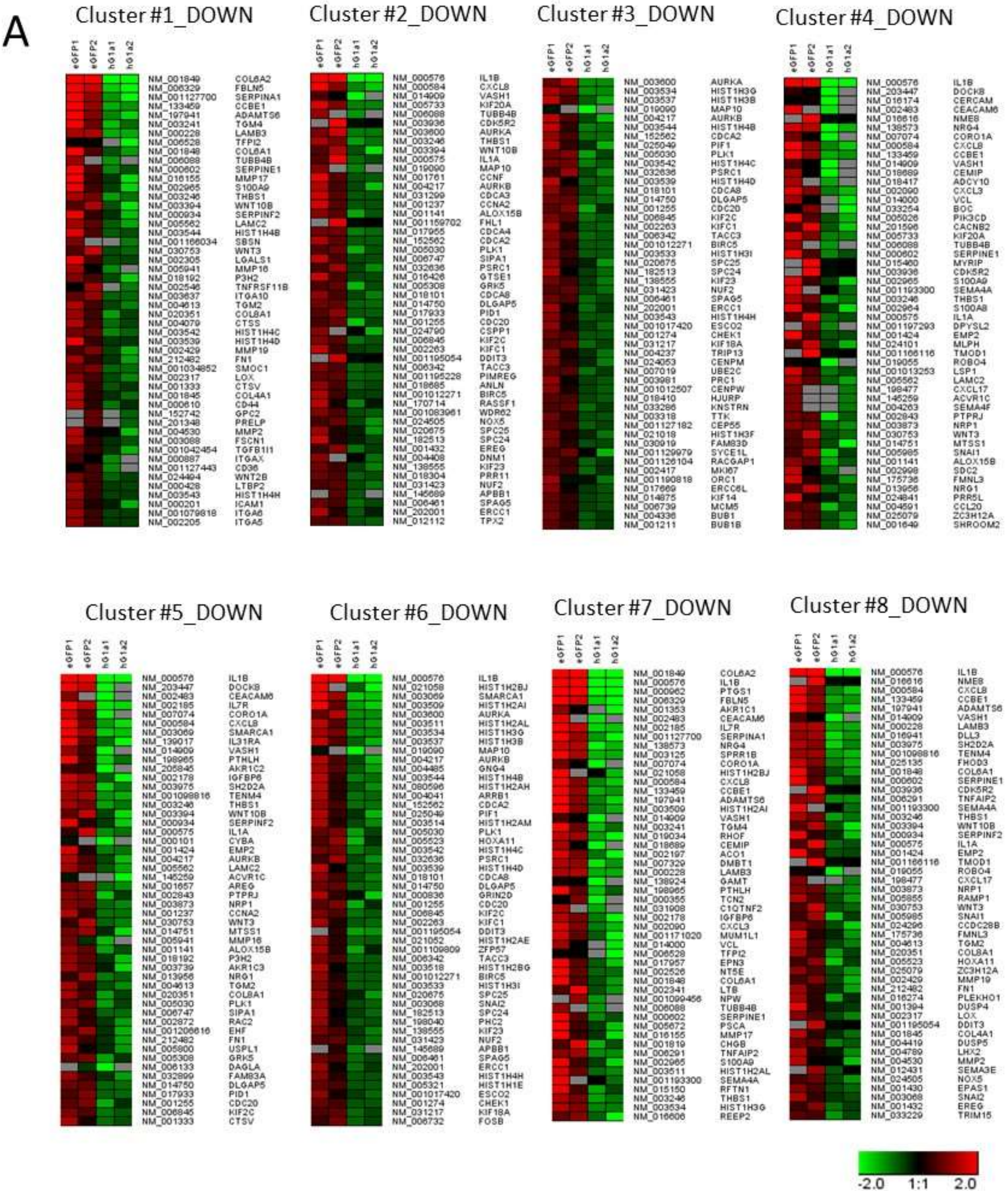

Supplementary Figure S4B: Heat maps of significantly DOWN-regulated gene clusters:

B

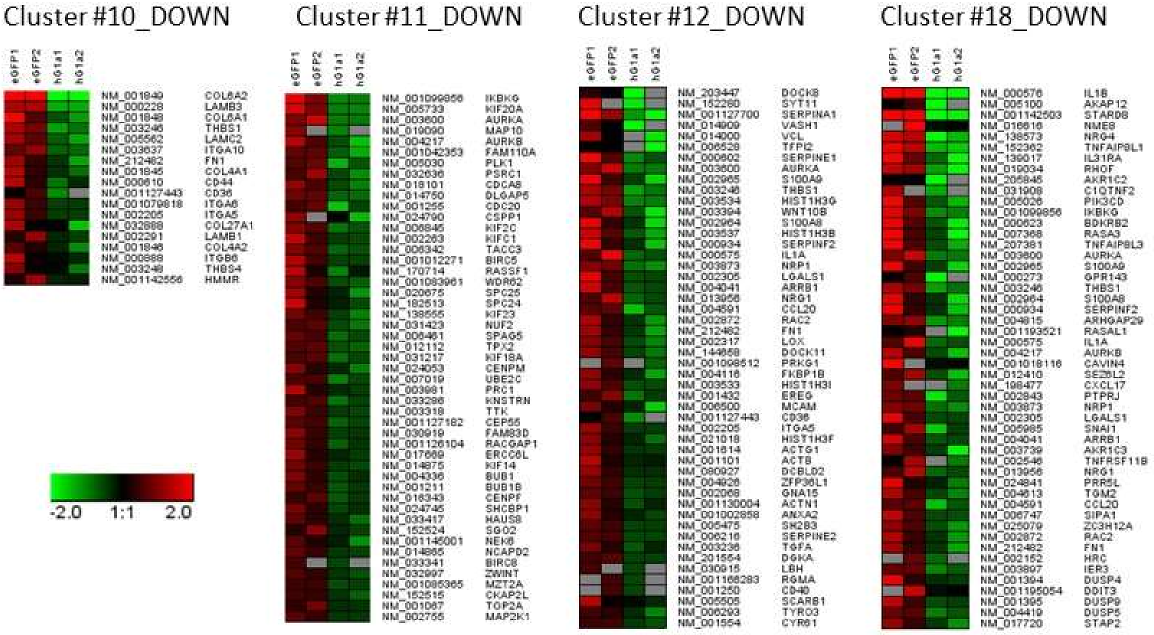

Supplementary Figure S5: Validation of RNA-seq by qPCR:

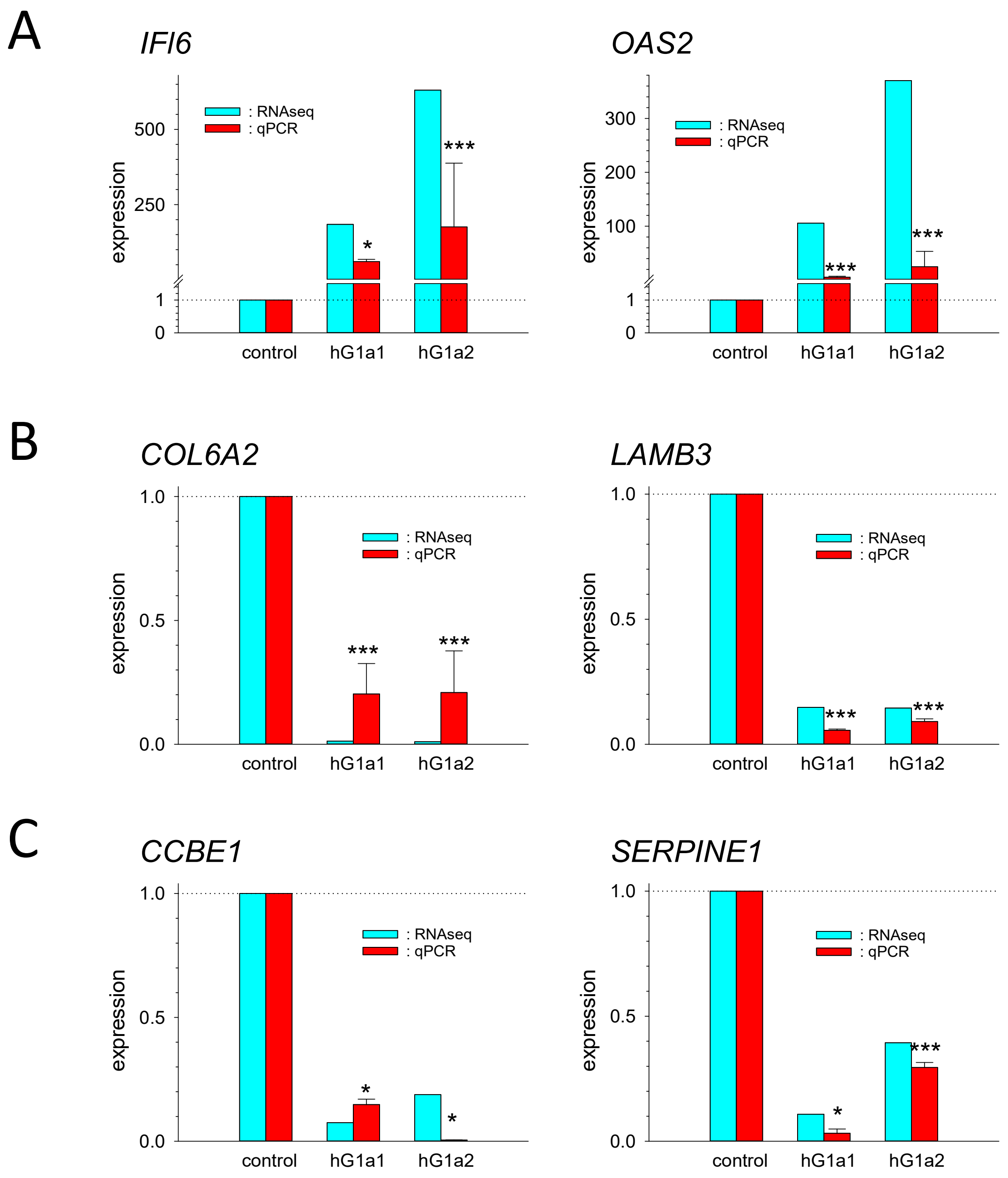

RNA-seq expression of six genes, selected from the three most important clusters was confirmed by independent qPCR (YWHAZ was used as reference for qPCR; see supplementary Table S2 for primer sequences). Expression level of the gene of interest in the libraries derived from GIRK1 overexpressors was normalized to one of the control libraries and are shown as fold change on the y-axis (eGFP1 was used as control for hG1a1 and eGFP2 as control for hG1a2, respectively). A high correlation between RNA-seq and qPCR expression levels could be detected. All genes were differentially expressed in comparison to the reference (*, (***): p-value is statistically significantly different at the <0.05, (0.001) level). **(A):** Gene cluster IFγ, **(B):** Gene cluster ECM and **(C):** gene cluster migration.

Supplementary Table S1: Key resource table of reagents

**Key resource table of reagents**			
**Product designation**	**Source or reference**	**Identifiers**	**Additional information**
L-Glutamine solution	Merck	G7513 100ML	200 mM
4x Laemmli buffer	BioRad	1610747	
7-AAD Viability Staining Solution	BD Biosciences	00-6993-50	
Acetic acid	Merck	1000632500	100%
AseI	NEB	R0526S	
BamHI	Promega	R6021	
BSA	Roth	3854.2	
Cell counting slides	Biozym	872010	
CHEMICON ECM Cell Adhesion Array Kit	MERCK	ECM540	
Cholera Toxin	Merck	C8052-.5MG	
Clean-Blot IP detection reagent	ThermoFisher	21230	
Click-iT EdU Pacific Blue Flow Cytometry Assay Kit	ThermoFisher	C10418	
complete Mini Protease inhibitor Cocktail	Merck	11836170001	
Costar 6-well Multiple Well Plates	Corning Inc.	3506	
crystal violet	Merck	C0775	0,10%
Difco TM Skim Milk	BD Biosciences	232100	
ECL Select Western Blotting Detection Reagent	MERCK	GERPN2235	

Product designation	Source or reference	Identifiers	Additional information
EdU	ThermoFisher	C10418	
FBS (Fetal Bovine Serum)	Merck	F7524-500ML	Lot No 025M3355
Frackelton buffer	-	-	10 mM Tris, 50 mM NaCl, 30 mM $Na_4P_2O_7$, 50 mM NaF, 200 mg/L Triton X-100, pH 7,1
G418 disulfate salt	Merck	A1720-1G	
GIRIK1 N-T	Kurt Schmidt	N/A	MK4
GIRK1	Abcam	119246	RRID:AB_10903846
GIRK1 C-T	Alomone Labs	APC-005	RRID:AB_2040113
Hard Shell 480 PCR 96 Multiwell plates	BioRad	4 729 692 001	
Immobilon-P PVDF Membrane	Merck	IPVH07850	
Ion AmpliSeq Transcriptome Human Gene Expression Kit	ThermoFisher	A26325	
Ion PI Chip Kit	ThermoFisher	A26771	
Kanamycin	MERCK	K0254-20ML	
Laemmli Sample Buffer	BioRad	1610747	
Lipofectamine 2000	ThermoFisher	11668027	
Matrigel	BD Biosciences	356237	
MCF10A	ATCC	ATCC® CRL-10317™	
MCF7	ATCC	ATCC® HTB-22™	
MEBM (Mammary Epithelial Cell Growth Basal Medium)	Lonza	CC-3151	

**Product designation**	**Source or reference**	**Identifiers**	**Additional information**
MEM (Minimum Essential Medium Eagle)	Merck	M2279-500ML	
Methanol	Roth	082.1	100%
NheI	Promega	R6501	
PBS, pH 7.4	ThermoFisher	10010-015	
Penicillin-Streptomycin	MERCK	P0781-100ML	
Peroxidase Goat Anti-Rabbit	Jackson Immuno Research	111-035-144	RRID:AB_2307391
Pierce™ Protein A Agarose	ThermoFisher	20333	
Pipette tip 200µl yellow	SARSTEDT	70.760.012	
pIRES2eGFP	Clontech	6029-1	
Ponceau S	Merck	P7170	
Protein A Agarose	ThermoFisher	20333	
QIA shredder spin column	QIAGEN	79645	
qPCR QuantiFast SYBR Green PCR Kit	QIAGEN	204054	
QuantiTect Rev. Transcription Kit	QIAGEN	205311	
RNeasy Mini Kit	QIAGEN	74104	
SingleQuots Supplements	Lonza	CC-4136	
Sodium Pyruvate	ThermoFisher	11360039	100 mM
Spectra Multicolor Broad Range Protein Ladder	ThermoFisher	26634	

Product designation	Source or reference	Identifiers	Additional information
SuperScript VILO cDNA Synthesis Kit	ThermoFisher	11754050	
SYBR Green PCR Kit	QIAGEN	204054	
T75 Flask	Sarstedt	83.3911.002	
TBST	-	-	200 mM/L TRIS<br>1400 mM/L NaCl
TC Dishes 35	Sarstedt	83.3900	
Tissue Culture Plate, 24 well	FALCON	353847	
Transfer Buffer	ThermoFisher	BT0006	

Supplementary Table S2: primers used for PCR

Primer designation	Sequence
YWHAZ-f:	5´ -ACTTTTGGTACATGTGGCTTCAA-3´
YWHAZ-r:	5´ -CCGCCAGGACAAACCAGTAT-3´
GAPDH-f:	5′-ATGGGGAAGGTGAAGGTCG-3′
GAPDH-r:	5′-GGGGTCATTGATGGCAACAATA-3′
qPCR-GIRK1a-f:	5′-GTGGAAACAACTGGGATGAC-3′
qPCR-GIRK1a-r:	5′-GTTGCATGGAACTGGGAGTA-3′
plasmid-GIRK1a-f:	5´-TTAGCTAGCATGTCTGCACTCCGAAGGAAATTTG-3´
plasmid-GIRK1a-r:	5´-TTAGGATCCTTATGTGAAGCGATCAGAGTT C-3´
qPCR-LAMB3B-f	5´-CTTTGACCCCGCTGTGTTTG-3´
qPCR-LAMB3B-r:	5´-CTGACACCGCTCACAGTTCT-3´
qPCR-COL6A2-f:	5´-GCAGAGCTGTCCTTCGTGT-3`
qPCR-COL6A2-r:	5´-TGGGTACCACGTTCTCGTT-3´
qPCR-IL1B-f:	5´-TGAGCTCGCCAGTGAAATGA-3`
qPCR-IL1B-r:	5´-AGATTCGTAGCTGGATGCCG-3`
qPCR-SERPINE1-f:	5´-GCCAGTGGAAGACTCCCTTC-3´
qPCR-SERPINE1-r:	5´-GTGCTGCCGTCTGATTTGTG-3´
qPCR-CCBE1-f:	5´-GGACACACGTGGACCTACAG-3´
qPCR-CCBE1-r:	5´-GTCGTCGCGATTTTGCTCTC-3´
qPCR-IFI6-f:	5´-GGTGAGAATGCGGGTAAGGAT-3´
qPCR-IFI6-r:	5´-AGGTCAGGGCCTTCCAGAAC-3´
qPCR-OAS2-f:	5´-CGTTGGTGTTGGCATCTTCTG-3´
qPCR-OAS2-r:	5´-TGCATTGTCGGCACTTTCCA-3´

Supplementary Table S3: List of significantly regulated clusters as detected by DAVID

UP-regulated						
Cluster No.	***Enrichement Score (ES)***	***p=(10^{-ES})***	***Benjamini-Hochberg***	***FDR***	***Prototyp_Name***	***also***
1	7.611970358	2.4436E-08	2.65E-16	9.24E-17	GO:0034340~response to type I interferon	GO:0071345~cellular response to cytokine stimulus
2	3.678708775	2.0955E-04	2.63E-09	4.58E-09	GO:0045087~innate immune response	GO:0001817~regulation of cytokine production
3	3.128065629	7.4462E-04	2.83E-04	1.56E-03	hsa05168:Herpes simplex infection	hsa05160:Hepatitis C
DOWN-regulated						
Cluster No.					Prototyp_Name	also
1	5.994629397	1.0124E-06	1.25E-06	1.09E-05	GO:0031012~extracellular matrix	hsa04512:ECM-receptor interaction
2	5.518649804	3.0294E-06	1.19E-06	3.60E-07	GO:0000280~nuclear division	GO:0051301~cell division
3	5.504568335	3.1292E-06	2.00E-05	3.03E-05	GO:0000819~sister chromatid segregation	GO:0007059~chromosome segregation
4	5.428137424	3.7313E-06	1.21E-05	1.47E-05	GO:0030335~positive regulation of cell migration	GO:0006935~chemotaxis
5	4.478903449	3.3197E-05	5.09E-05	2.47E-04	GO:0008283~cell proliferation	GO:0008284~positive regulation of cell proliferation
6	4.443323983	3.6031E-05	8.18E-10	1.80E-09	GO:0044815~DNA packaging complex	GO:0000786~nucleosome
7	4.274924673	5.3098E-05	2.18E-05	3.35E-04	GO:0044421~extracellular region part	GO:0005576~extracellular region
8	4.225635365	5.9479E-05	2.69E-05	5.71E-05	GO:0001568~blood vessel development	GO:0001944~vasculature development
9	3.400777675	3.9739E-04	0.002764337	0.034363623	GO:0001704~formation of primary germ layer	GO:0001706~endoderm formation
10	3.356937953	4.3960E-04	4.63E-04	6.87E-03	hsa04512:ECM-receptor interaction	hsa04510:Focal adhesion
11	3.26593354	5.4208E-04	2.00E-05	3.03E-05	GO:0000819~sister chromatid segregation	GO:0000070~mitotic sister chromatid segregation
12	3.138542676	7.2687E-04	0.006853849	0.108241341	GO:0009611~response to wounding	GO:0042060~wound healing
13	3.061209779	8.6854E-04	0.002677864	0.032475661	GO:0007088~regulation of mitotic nuclear division	GO:0051783~regulation of nuclear division
14	2.702736206	1.9827E-03	0.061642788	2.304387456	GO:0030574~collagen catabolic process	GO:0044259~multicellular organismal macromolecule metabolic process
15	2.390350153	4.0705E-03	0.043967133	1.379243118	GO:0051781~positive regulation of cell division	GO:0045787~positive regulation of cell cycle
16	2.371547907	4.2506E-03	0.008902757	0.159649619	GO:0002687~positive regulation of leukocyte migration	GO:0097530~granulocyte migration
17	2.327843649	4.7006E-03	0.015561026	0.327171071	GO:0060707~trophoblast giant cell differentiation	GO:0009790~embryo development
18	2.316570367	4.8242E-03	0.006848572	0.110236555	GO:1902531~regulation of intracellular signal transduction	GO:0005085~guanyl-nucleotide exchange factor activity
19	2.231532319	5.8677E-03	0.02470726	0.604252092	GO:0007051~spindle organization	GO:0000022~mitotic spindle elongation
20	2.218123064	6.0517E-03	0.012299808	0.243313002	GO:0007186~G-protein coupled receptor signaling pathway	GO:0004872~receptor activity
21	2.156449856	6.9751E-03	0.038651025	1.092183278	GO:0031589~cell-substrate adhesion	GO:0010810~regulation of cell-substrate adhesion
22	2.128847423	7.4328E-03	0.033701725	2.672854827	GO:0030864~cortical actin cytoskeleton	GO:0030863~cortical cytoskeleton
23	2.069397176	8.5232E-03	0.002764337	0.034363623	GO:0001704~formation of primary germ layer	GO:0007369~gastrulation
24	2.007981647	9.8179E-03	0.006848572	0.110236555	GO:1902531~regulation of intracellular signal transduction	GO:0023051~regulation of signaling
25	1.905711994	1.2425E-02	0.081254757	0.977197772	GO:0008092~cytoskeletal protein binding	GO:0030029~actin filament-based process
26	1.854749417	1.3972E-02	0.0478295	1.531717555	GO:0048608~reproductive structure development	GO:0061458~reproductive system development
27	1.851650373	1.4072E-02	1.39E-04	1.01E-03	GO:0051290~protein heterotetramerization	GO:0034723~DNA replication-dependent nucleosome organization
28	1.835203732	1.4615E-02	0.015773628	0.346064714	GO:0007346~regulation of mitotic cell cycle	GO:1902749~regulation of cell cycle G2/M phase transition
29	1.794551181	1.6049E-02	0.294675707	8.884232497	GO:0005539~glycosaminoglycan binding	GO:0008201~heparin binding
30	1.765752957	1.7149E-02	0.008298175	0.141211424	GO:0007350~blastoderm segmentation	GO:0048846~axon extension involved in axon guidance
