# Supplementary material for: GIRK1 triggers multiple cancer-related pathways in the benign mammary epithelial cell line MCF10A
Source: Sci Rep. 2019 Dec 17;9:19277. doi: 10.1038/s41598-019-55683-w (PMC6917815; doi:10.1038/s41598-019-55683-w)
Supplement: Supplementary file 2 — Supplementary information2 [file 41598_2019_55683_MOESM2_ESM.docx]

**ALGov9 macro (for wound healing)**

**// This macro is copyrite of Dr Trevor DeVaney Feb 2017**

**//**

**//setBatchMode(true);**

**//========================================================**

**//copywrite declaration**

**//========================================================**

**string="This macro is copyrite of Dr Trevor DeVaney Feb 2017 \n This macro is for detecting the area covered by cells in sequences of images\n Timelapse sequences for monitoring growth";**

**Dialog.create("Covered area assesment macro");**

**Dialog.addMessage(string);**

**Dialog.show();**

**string="In the composite images \n \n Red is the areas detected\n Green is the area included in the measurement\n Yellow is both detected and measured.";**

**Dialog.create("Covered area assesment macro");**

**Dialog.addMessage(string);**

**Dialog.show();**

**//========================================================**

**//Default parameters**

**//========================================================**

**inv=1;**

**hole=1;**

**sl=10000;**

**su="Infinity";**

**cl= 0.001;**

**cu=0.999;**

**v=30;**

**m=6;**

**sti=1;**

**imi=1**

**mni=5000**

**sc=100**

**fftlow=1**

**ffthigh=40**

**//========================================================**

**//Dialog import parameters**

**//========================================================**

**Dialog.create("Choose image import selction parameters");**

**Dialog.addCheckbox("Invert analysis",inv);**

**Dialog.addSlider("Starting image number", 1,50000, sti);**

**Dialog.addSlider("Image increment", 1, 20000000, imi);**

**Dialog.addSlider("Maximum number of images to import", 5, 5000, mni);**

**Dialog.addSlider("Image scale", 0, 100, sc);**

**Dialog.show();**

**inv = Dialog.getCheckbox();**

**sti = Dialog.getNumber();**

**imi = Dialog.getNumber();**

**mni = Dialog.getNumber();**

**sc = Dialog.getNumber();**

**//========================================================**

**//close log window if open**

**//========================================================**

**if (isOpen("Log")==true) {**

**selectWindow("Log");**

**run ("Close");**

**}**

**//========================================================**

**//open new log window for protocol**

**//========================================================**

**print("//========================================================\n" );**

**run("Set Measurements...", "area mean min median redirect=None decimal=3");**

**run("Input/Output...", "jpeg=100 gif=-1 file=.txt copy_column copy_row save_column save_row");**

**//========================================================**

**//directory selection**

**//========================================================**

**j=0;**

**while (j<1){**

**print("sample directory selection");**

**dir = getDirectory("Select a Data Directory");**

**j=getBoolean("Is the sample directory correct ? \n"+dir );**

**print("sample directory is = "+dir );**

**}**

**//========================================================**

**//results directory selection**

**//========================================================**

**date="results";**

**print("results directory is = " +dir+"\\"+date);**

**if (File.isDirectory(dir+"\\"+date)==false){**

**print("directory absent, creating!");**

**File.makeDirectory(dir+"\\"+date);**

**}**

**if (File.isDirectory(dir+"\\"+date)==true){**

**print("success "+dir+"\\"+date);**

**}**

**//========================================================**

**//iterative file open routine**

**//========================================================**

**print("//========================================================\n" );**

**//======================================================**

**// list is an array with the file names in it**

**//======================================================**

**list = getFileList(dir);**

**//======================================================**

**// recursively call the list of files**

**//======================================================**

**for (i=0; i<list.length; i++) {**

**print("next directory i="+i);**

**print("name is "+list[i]);**

**print("processing "+dir+list[i]);**

**//======================================================**

**// opening file**

**//======================================================**

**if (File.isDirectory(dir+"\\"+date)==true){**

**i=i+1;**

**}**

**//======================================================**

**// import trans image sequences**

**//======================================================**

**print("Importing image sequence from "+dir+list[i]);**

**run("Image Sequence...", "open=["+dir+"\\"+list[i]+"\\] number="+mni+" starting="+sti+" increment="+imi+" scale="+sc+" file=[] or=[] sort");**

**//open(dir+list[i]);**

**run("8-bit");**

**//========================================================**

**//repeat loop**

**//========================================================**

**j=0;**

**while (j<1){**

**//========================================================**

**//choose parameters dialog**

**//========================================================**

**Dialog.create("Choose selction parameters");**

**Dialog.addCheckbox("Fill holes",hole);**

**Dialog.addSlider("Size lower limit", 0, 5000000, sl);**

**Dialog.addSlider("Size upper limit", 1, 2000000, su);**

**Dialog.addSlider("Circularity lower limit", 0.0, 1.0, cl);**

**Dialog.addSlider("Circularity upper limit", 0.0, 1.0, cu);**

**Dialog.addSlider("Detection (Variance)", 1, 100, v);**

**Dialog.addSlider("Smoothing (Median)", 1, 100, m);**

**Dialog.addSlider("FFT Low Bandpass", 1, 1000, fftlow);**

**Dialog.addSlider("FFT High Bandpass", 1, 1000, ffthigh);**

**Dialog.show();**

**hole = Dialog.getCheckbox();**

**sl = Dialog.getNumber();**

**su = Dialog.getNumber();**

**cl = Dialog.getNumber();**

**cu = Dialog.getNumber();**

**v = Dialog.getNumber();**

**m = Dialog.getNumber();**

**fftlow = Dialog.getNumber();**

**ffthigh = Dialog.getNumber();**

**if(su==2000000){**

**su="Infinity";**

**}**

**//========================================================**

**//calculation**

**//========================================================**

**//========================================================**

**//duplicate image**

**//========================================================**

**run("Duplicate...", "title=[copy2_"+list[i]+"] duplicate range=1-500");**

**run("Duplicate...", "title=[copy_"+list[i]+"] duplicate range=1-500");**

**//========================================================**

**//FFT bandpass**

**//========================================================**

**run("Bandpass Filter...", "filter_large="+ffthigh+" filter_small="+fftlow+" suppress=None tolerance=5 autoscale saturate process");**

**//========================================================**

**//Variance analysis**

**//========================================================**

**run("8-bit");**

**run("Variance...", "radius="+v+" stack");**

**//========================================================**

**//threshold image**

**//========================================================**

**setAutoThreshold("Mean dark");**

**setOption("BlackBackground", false);**

**run("Convert to Mask", "stack");**

**//========================================================**

**//median analysis**

**//========================================================**

**run("Median...", "radius="+m+" stack");**

**//========================================================**

**//Measurement inversion**

**//========================================================**

**if (inv == true) {**

**run("Invert", "stack");**

**}**

**//========================================================**

**//Hole inclusion**

**//========================================================**

**if (hole == true) {**

**run("Analyze Particles...", "size="+sl+"-"+su+" circularity="+cl+"-"+cu+" show=Masks display clear include summarize stack");**

**} else {**

**//========================================================**

**//Hole exclusion**

**//========================================================**

**run("Analyze Particles...", "size="+sl+"-"+su+" circularity="+cl+"-"+cu+" show=Masks display clear summarize stack");**

**}**

**print("//========================================================\n" );**

**//========================================================**

**//image viewing**

**//========================================================**

**window="Mask of copy_"+list[i];**

**selectWindow(window);**

**run("Grays");**

**run("Subtract...", "value=170 stack");**

**selectWindow("copy_"+list[i]);**

**run("Subtract...", "value=170 stack");**

**run("Merge Channels...", "red=[copy_"+list[i]+"] green=[Mask of copy_"+list[i]+"] blue=*None* gray=[copy2_"+list[i]+"] create keep");**

**//========================================================**

**// repeat selection ?**

**//========================================================**

**waitForUser("Check the results and press OK when finished");**

**j=getBoolean("Were the parameters correct ? \n Cancel cancels the macro\n No requires new parameter selection\n Yes saves the data and continues with the next file");**

**selectWindow("Composite");**

**if(j==true){**

**a=lengthOf(list[i]);**

**name=substring(list[i],0,a-7);**

**print("name= "+name);**

**print("Composite saved to "+dir+date+"\\Composite_of_"+name+".tif");**

**saveAs("Tiff", dir+date+"\\Composite_of_"+name);**

**run("Close");**

**}else{**

**selectWindow("Composite");**

**close();**

**selectWindow("Mask of copy_"+list[i]);**

**close();**

**selectWindow("copy_"+list[i]);**

**close();**

**selectWindow("copy2_"+list[i]);**

**close();**

**if (isOpen("Summary of copy_"+list[i])==true) {**

**selectWindow("Summary of copy_"+list[i]);**

**run("Close");**

**}**

**if (isOpen("Results")==true) {**

**selectWindow("Results");**

**run("Close");**

**}**

**}**

**}**

**//========================================================**

**// write the parameter settings**

**//========================================================**

**print("//========================================================\n" );**

**print("Parameters for = "+list[i]);**

**print("Invert analysis = "+inv);**

**print("Fill Holes = "+hole);**

**print("Lower size = "+sl);**

**print("Upper size = "+su);**

**print("Lower circularity = "+cl);**

**print("Upper circularity = "+cu);**

**print("Detection = "+v);**

**print("Upper Smoothing = "+m);**

**print("Starting image number = "+sti);**

**print("Image increment = "+imi);**

**print("Maximum number of images to import = "+mni);**

**print("Image scale = "+sc);**

**print("//========================================================\n" );**

**//========================================================**

**// close results window**

**//========================================================**

**if (isOpen("Results")==true) {**

**selectWindow("Results");**

**saveAs("Text", dir+date+"\\Results_"+name+".txt");**

**run("Close");**

**}**

**//========================================================**

**// close Summary window**

**//========================================================**

**if (isOpen("Summary of copy_"+list[i])==true) {**

**selectWindow("Summary of copy_"+list[i]);**

**saveAs("Text", dir+date+"\\Summary of "+name+"_.txt");**

**run("Close");**

**}**

**//========================================================**

**// close image windows**

**//========================================================**

**while (nImages()>0) {**

**selectImage(nImages());**

**run("Close");**

**}**

**//========================================================**

**// close log window**

**//========================================================**

**if (isOpen("Log")==true) {**

**selectWindow("Log");**

**saveAs("Text", dir+date+"\\Log"+name+".txt");**

**run("Close");**

**}**

**//========================================================**

**//end directory processing**

**//========================================================**

**}**

**print("//========================================================\n" );**

**print("end of processing");**

**print("//========================================================\n" );**

**//========================================================**

**// close log window**

**//========================================================**

**if (isOpen("Log")==true) {**

**selectWindow("Log");**

**saveAs("Text", dir+date+"\\Log.txt");**

**run("Close");**

**}**

**print("//========================================================\n" );**

**print("End of program");**

**print("//========================================================\n" );**
